# Biomimetic in situ tracheal microvascularization for segmental tracheal reconstruction in one‐step

**DOI:** 10.1002/btm2.10534

**Published:** 2023-05-03

**Authors:** Fei Sun, Zhiming Shen, Boyou Zhang, Yi Lu, Yibo Shan, Qiang Wu, Lei Yuan, Jianwei Zhu, Shu Pan, Zhihao Wang, Cong Wu, Guozhong Zhang, Wenlong Yang, Xiangyu Xu, Hongcan Shi

**Affiliations:** ^1^ Clinical Medical College Yangzhou University Yangzhou China; ^2^ Institute of Translational Medicine, Medical College Yangzhou University Yangzhou China; ^3^ Jiangsu Key Laboratory of Integrated Traditional Chinese and Western Medicine for Prevention and Treatment of Senile Diseases Yangzhou University Yangzhou China; ^4^ Department of Thoracic Surgery The First Affiliated Hospital of Soochow University Suzhou China

**Keywords:** endothelial progenitor cells, functionalization, microvascularization, tissue engineering, tracheal transplantation

## Abstract

Formation of functional and perfusable vascular network is critical to ensure the long‐term survival and functionality of the engineered tissue tracheae after transplantation. However, the greatest challenge in tracheal‐replacement therapy is the promotion of tissue regeneration by rapid graft vascularization. Traditional prevascularization methods for tracheal grafts typically utilize omentum or muscle flap wrapping, which requires a second operation; vascularized segment tracheal orthotopic transplantation in one step remains difficult. This study proposes a method to construct a tissue‐engineered tracheal graft, which directly forms the microvascular network after orthotopic transplantation in vivo. The focus of this study was the preparation of a hybrid tracheal graft that is non‐immunogenic, has good biomechanical properties, supports cell proliferation, and quickly vascularizes. The results showed that vacuum‐assisted decellularized trachea‐polycaprolactone hybrid scaffold could match most of the above requirements as closely as possible. Furthermore, endothelial progenitor cells (EPCs) were extracted and used as vascularized seed cells and seeded on the surfaces of hybrid grafts before and during the tracheal orthotopic transplantation. The results showed that the microvascularized tracheal grafts formed maintained the survival of the recipient, showing a satisfactory therapeutic outcome. This is the first study to utilize EPCs for microvascular construction of long‐segment trachea in one‐step; the approach represents a promising method for microvascular tracheal reconstruction.

## 1 INTRODUCTION

Tracheal transplantation remains an unresolved clinical challenge worldwide. This is primarily owing to the lack of ideal tracheal substitutes and the obstruction of graft vascularization, which is caused by special anatomical structures.^1^, ^2^ Longer segment trachea (more than 50% in adults or 30% in children) lesions caused by tracheal tumors, stenosis, trauma, and softening cause added complications in surgical operations. In such situations, end‐to‐end anastomosis fails, and serious complications, such as anastomotic leakage and tracheal rupture, are caused owing to high anastomotic tension.^3^ Tracheal transplantation with a substitute is then an effective treatment method to achieve healthy airway repair.^4^ Clinical tracheal transplantation using bioprosthesis,^5^ allograft,^6^, ^7^, ^8^ or autologous tissue reconstruction^9^, ^10^, ^11^ has been performed; however, but the effects have been highly unsatisfactory. The main shortcomings are as follows: (1) owing to poor biocompatibility and rigid structure, the bioprosthesis can easily cause hyperplasia of granulation tissue and tracheal stenosis; (2) long‐term use of immunosuppressive agents is required by patients for the allograft to be immunogenic, and a lack of effective blood supply to the graft leads to necrosis; (3) reconstruction of the structure of the bionic native trachea through autologous tissue is challenging and may lead to its abnormal functioning, increasing the possibility of surgical trauma.^12^

Tissue‐engineering technology combines living cells with scaffolds and has the potential to construct highly bionic functionalized tracheal substitutes.^13^ However, this technique still has not achieved the desired effect in clinical practice owing to postoperative complications such as softening, collapse, lumen stenosis, and blood circulation disorders of grafts.^14^, ^15^, ^16^ A key scientific issue with regenerative materials is the need to provide tissue‐specific biochemical and biomechanical properties, while facilitating cell proliferation and migration for functional graft formation and the repair of normal tissue. In addition, the development of a suitable biomaterial that contains angiogenesis‐inducing substances, that can promote angiogenic factor secretion and endothelial differentiation of seeded cells, is a meaningful way to promote the vascularization in tissue engineering.^17^ From the perspective of tissue composition and structure, the decellularized extracellular matrix (dECM) from natural tissue is an ideal biomaterial that preserves complex molecular components and complete tissue structure.^18^, ^19^ Our recent study showed that vacuum‐assisted decellularized trachea (VADT) exhibited extremely low immunogenicity and did not induce an inflammatory response when transplanted in vivo.^20^ However, insufficient biomechanical properties are a major limitation of these natural materials. Thus, constructing tissue structures with the desired biological functions and biomechanical properties from existing biomaterials remains a challenge. One approach involves a hybrid structure of synthetic and natural materials: synthetic materials provide suitable mechanical properties and a complete physical structure for tissue remodeling, and natural materials provide a suitable biochemical microenvironment for cell adhesion, proliferation, and differentiation.^21^, ^22^ Polycaprolactone (PCL) has been widely demonstrated to have excellent biocompatibility, suitable mechanical properties, and printability for 3D printing a tracheal scaffold.^23^, ^24^, ^25^ However, PCL is unfavorable for cell affinity, angiogenesis, and tissue regeneration when used alone, owing to its hydrophobicity.^26^ Based on these properties, a VADT‐PCL hybrid scaffold is expected to provide both biomechanical and stable cell affinity for tracheal grafts.

Foremost among the main challenges of all approaches in regenerative medicine is the ability to construct the vasculature of engineered tissues and translate them into a clinical model.^27^ The goals of vascularized construction include ensuring that the entire graft is perfused, and adequate nutrients and oxygen are received for long‐term survival. In addition, a well‐developed vascular network, such as capillaries and microvessels, is necessary for neonatal tissue.^28^ The blood supply to the trachea depends on small blood vessels that infiltrate between the cartilage rings and the mucosal layer of the inner wall to provide a segmental blood supply. Vascularization of the tissue‐engineered trachea is crucial for the survival of tracheal epithelial cells. More importantly, the vascularized trachea also plays an important role in resisting infection and reducing the necrosis and stenosis of the graft.^29^, ^30^ Neovascularization can occur via angiogenesis or vasculogenesis.^31^ Angiogenesis is the formation of new blood vessels by sprouting or by splitting based on pre‐existing vessels.^32^ By contrast, vasculogenesis refers to the process in which endothelial precursor cells aggregate, differentiate, and reorganize during the embryonic period, involving the formation of new blood vessels from bone marrow (BM) derived endothelial progenitor cells (EPCs).^33^ Owing to their high potential for in vitro expansion and acquisition of the mature endothelial cell phenotype, EPCs have gained considerable interest as an alternative source of endothelial cells (ECs), as well as seed cells that effectively promote neovascularization when transplanted in vivo.^34^ However, the role of BM‐EPCs in promoting vascularization in tissue‐engineered tracheas in vivo remains unknown.

Therefore, this study proposes the development of a hybrid tracheal graft that meets the following clinical needs: (1) low immunogenicity, good biocompatibility, and provision of a good microenvironment for cell growth; (2) good biomechanical properties to keep the lumen unobstructed; and (3) ability to form a microvascular network in a short time to promote the formation of functional grafts and long‐term survival of the recipient. In this study, we prepared VADT scaffolds that exhibited low immunogenicity and supported cell growth and angiogenesis. Subsequently, PCL macroporous mesh stents that matched the diameter of the VADT were prepared using 3D‐printing technology with good compression and resilience, and they were used to construct hybrid grafts in combination with the VADT that matched the biomechanical properties of the native trachea. Next, the EPCs were separated from BM and seeded on the surface of the hybrid grafts in vitro. Finally, Matrigel loaded with EPCs and vascular endothelial growth factor (VEGF) was used as a coating of the hybrid grafts during surgery for the orthotopic transplantation of segment tracheae, to promote the formation of vascularization and the functionalization of the grafts in vivo.

## 2 MATERIALS AND METHODS

### 2.1 Animal care and ethics statement

A total of 70 adult female New Zealand white rabbits weighing 2.5–3.2 kg were used as donors (*n* = 40) and recipients (*n* = 30). Five 3‐week‐old female New Zealand white rabbits weighing 0.2–0.3 kg were used for donors of EPCs, and nine male Wistar rats weighing 0.25–0.3 kg acted as embedding recipients in this study; they were purchased from the Laifu Farm in the Pukou District of Nanjing City (SYXK 2019‐0005). The protocol was reviewed and approved by the Experimental Animal Welfare Ethics Committee of Yangzhou University (No. 202202130). Representatives of the animal‐care staff examined the study animals during all stages of the project. All animals were handled in accordance with standards published by the National Academy of Sciences Act in the “Guidelines for the Care and Use of Laboratory Animal” Eighth Edition (2011).

### 2.2 Preparations and characterization of VADT

The VADT was prepared in a vacuum (−0.96 MPa) created by a microcomputer negative vacuum pump (Fujiwara Tools Co., Ltd., Taizhou, China) and processed in a shaking incubator at 80 rpm according to a previously described technique.^20^ The native tracheal tissues (approximately 5‐cm long segments) that were obtained from adult New Zealand white rabbits were incubated in sterile distilled water at 4°C for 24 h, and then were incubated in a detergent solution containing 0.25% Triton X‐100 (Biofroxx, Einhausen, Germany) and 0.25% sodium deoxycholate (Sigma, CA, USA) at 37°C for 24 h. The segments were then washed in sterile distilled water three times for 30 min, and the scaffolds were subjected to enzymatic digestion with 1 kU/mL DNAse (Sigma, CA, USA) and 2 U/mL RNAse (Biofroxx, Einhausen, Germany) in 1 M NaCl at 37°C for another 24 h. Finally, the decellularized tracheal segments were stored in phosphate‐buffered saline (PBS) containing 1% antibiotic and antimycotic solution at 4°C.

To determine the decellularization efficacy, hematoxylin and eosin (H&E) staining (Solarbio, Beijing, China) was used to evaluate the morphology, Masson's trichrome (MT) staining (Solarbio, Beijing, China) was used for fibrin‐collagen visualization, and Safranin O Fast Green (SOG) staining (Solarbio, Beijing, China) was performed to evaluate the glycosaminoglycan (GAG) content. The sections were then observed by optical microscopy (BX41TF‐5, Olympus, Japan).

Sections were also stained for the immunofluorescence (IF) analysis of major histocompatibility complex class I (MHC‐I) (MCA810GA, Bio‐Rad, Hercules, USA), MHC‐II (MCA811GA, Bio‐Rad, Hercules, USA), and 4′‐6‐diamidino‐2‐phenylindole (DAPI; KeyGEN, Nanjing, China) to detect immune components and nuclear material. An IF of type II collagen (1320, Southern Biotech, USA) was performed to evaluate collagen expression. Slides were analyzed by IF microscopy (EVOS FL; Invitrogen, USA).

Quantitative DNA analysis was performed using the Genomic DNA Purification Kit (Shenergy Biocolor, Shanghai, China) and a microplate reader (Epoch, BioTek, USA). The total collagen and sulfated GAG contents in each group of tissues were quantified using the Total Collagen Assay Kit (QuickZyme, Netherlands) and the Blyscan sGAG Assay Kit (Biocolor, County Antrim, UK) according to the manufacturer's instructions, respectively.

### 2.3 Fabrication and characterization of 3D hybrid scaffolds

The 3D printed rotating shaft was customized to match the native trachea based on the dynamic CT image data of the New Zealand rabbit trachea. PCL (Mn = 80,000 g/mol, Sigma, CA, USA) was selected as the printing material; the barrel temperature was 90°C; the printing speed was 2 mm/s; the printing length was 10 or 50 mm; sinusoidal filling was set as the printer's fill mode with 10, 20, 30, or 40 fill lines per circle; and the printing was performed on a customized axis of rotation. Subsequently, the PCL‐10, PCL‐20, PCL‐30, and PCL‐40 tubular stents were fabricated on the rotating shaft using a 3D bioprinter (3D Bio‐architect SR, Regenovo, Hangzhou, China) corresponding to the 10, 20, 30, or 40 fill lines per circle samples, respectively. Scanning electron microscopy (SEM; S‐4800, Hitachi High‐Technologies Corporation, Tokyo, Japan) was performed to observe the apertures of the PCL stents. The PCL stents were guided by a sterile glass with a matched diameter and then implanted inside the VADT and to construct hybrid scaffolds. The biomechanical properties of the native, VADT, VADT/PCL‐10, VADT/PCL‐20, VADT/PCL‐30, and VADT/PCL‐40 samples (*n* = 3 for each condition) were evaluated using a universal tensile tester. The tension of the 5‐cm long tracheal graft segments was applied parallel to the long axis, while the compression of the 1‐cm long tracheal graft segments and the three‐point bending of the 5‐cm long tracheal graft segments were applied perpendicular to the long axis. A vernier caliper was used to measure the length, thickness, and outer and inner diameters of each sample. The load of displacement in 2.5 mm and the elastic modulus between 20% and 80% of max displacement were tested in the compressive mode; the load of displacement in 15 mm and the elastic modulus between 20% and 80% max load were tested in the three‐point bending mode; the load of breaking point and the elastic modulus between 20% and 80% max load were tested in the tensile mode.

The hybrid scaffolds of VADT/PCL‐20 were soaked in PBS at 37°C and the PBS was replaced every 24 h. The formula for calculating wet mass of the hybrid scaffolds loss is: *η* = ((*W*_*o*_ − *W*_*t*_)/*W*_*o*_) × 100%; *W*_*o*_ represents the initial weight of the scaffolds and *W*_*t*_ represents their weight after soaking time. Three scaffolds were collected from each time point (0, 7, 14, and 28 days). The mechanical properties of the VADT/PCL‐20 scaffolds were investigated by performing uniaxial mechanical tests on the degraded scaffolds. All the scaffolds were soaked in PBS at 37°C. After each time point (0, 14, and 28 days), degraded scaffolds were collected for uniaxial mechanical test with universal tester. The samples were loaded at a rate of 2 mm/min and the elastic modulus was measured.

### 2.4 Extraction and identification of EPCs

The EPCs were isolated and cultured using whole marrow differential adherence. Briefly, BM was isolated from the femurs of 3‐week‐old female rabbits and cultured in DMEM‐F12 medium containing 10% fetal bovine serum (FBS, Gibco, NY, USA) in an incubator (HERAcell 150i, Thermo Fisher, Waltham, USA) at 37°C in 5% CO_2_ for 20 h. Nonadherent cells were then collected and cultured in EBM‐2MV medium (Lonza, Basel, Switzerland). Subsequently, the medium was replaced every 2 days. The EPCs were harvested at the second passage (P2) and stained with the following fluorescence‐conjugated antibodies: CD31/APC (Bioss, Beijing, China), CD34/PE (Genetex, SC, USA), CD 44/PE‐Cy7 (Bioss, Beijing, China), and CD105/FITC (Genetex, SC, USA). The EPCs were also analyzed by IF staining with mouse anti‐CD31 (GeneTex, CA, USA), rat anti‐CD34 (GeneTex, CA, USA), and goat anti‐VEGFR2 (Biorbyt, Cambridge, UK) antibodies to confirm the endothelial phenotype after 24 h of culture.

### 2.5 Biocompatibility of the hybrid scaffolds

The biocompatibility of the hybrid scaffolds was evaluated as follows. First, a cell‐counting kit‐8 (CCK‐8, Biosharp, Hefei, China) assay was performed to examine the proliferation of EPCs on the scaffolds after 1, 3, and 5 days of culture. Next, live/dead staining (KeyGEN, Nanjing, China) was performed to examine the viability of the EPCs on the scaffolds after 24 h of culture. Finally, the microstructure of the cell‐scaffold composite was observed using SEM (GeminiSEM 300, Hitachi, Japan).

### 2.6 Chicken embryo chorioallantoic membrane assays

Chorioallantoic membrane (CAM) assays were used as in vivo models to evaluate the angiogenic properties of the bioengineered tracheae. As previously reported,^35^, ^36^ fertilized chicken eggs (Spaifrui, Jinan, China) were incubated at 37.8°C at constant humidity and randomly divided into three groups (*n* = 5 for each condition): native, VADT, and VADT + EPCs. On Day 8 of incubation, an approximately 2 cm diameter window was cut into the shell with small dissecting scissors to reveal the embryo and CAM vessels. The matrices of each group (2 × 2 mm) were then placed on the CAM between the branches of the blood vessels. The window was sealed with glass, and the eggs were returned to the incubator. CAMs were inspected and photographed daily for 4 days after the placement of the matrices.

### 2.7 In vitro tube formation assay

Matrigel (Cat. No. 356234, BD, Bedford, MA, USA) was placed on ice overnight, and added to 48‐well plates (100 μL/well) in three groups (three sub‐wells per group): VEGF, EPCs, and VEGF + EPCs. Subsequently, the Matrigel was incubated at 37°C for 1 h to form the gel. Next, VEGF (Cat. No. C083, Novoprotein, Shanghai, China; 200 ng), EPCs (2 × 10^4^/200 μL), and 200 ng VEGF and 2 × 10^4^/200 μL EPCs were added to achieve VEGF, EPCs, and VEGF + EPCs groups, respectively; then, the 48‐well plate was incubated at 37°C for 24 h in a cell incubator. The formation of tube‐like structures was observed by light microscopy (BX41TF‐5, Olympus, Japan).

### 2.8 In vivo neovascularization assay

To verify the in vivo angiogenic properties of the Matrigel loaded with VEGF and EPCs, it was embedded in Wistar rats. Matrigel was placed on ice overnight, and then added to 48‐well plates (100 μL/well) in three groups (three sub‐wells per group): VEGF, EPCs, and VEGF + EPCs. VEGF group was inoculated with VEGF 1 μg, EPCs group with 1 × 10^6^ EPCs, and VEGF + EPCs group with VEGF 1 μg and 1 × 10^6^ EPCs. Next, the Matrigel was incubated at 37°C for 1 h to form the gel and implanted subcutaneously in the abdomen of Wistar rats (*n* = 9). The grafts were obtained 7 days postoperatively for gross visualization and histological analysis.

### 2.9 Orthotopic transplantation of segment hybrid‐engineered trachea

#### 2.9.1 Experimental groups

From the above experimental results, the hybrid tracheal grafts with the best biomechanical properties and biocompatibility were selected for orthotopic transplantation experiments. Adult female New Zealand rabbits were selected as 1‐cm long segment tracheal transplantation subjects and divided into the following six groups (*n* = 5 for each condition): Group A, allogeneic tracheal transplantation; Group B, VADT scaffold transplantation; Group C, VADT + PCL grafts transplantation; Group D, VADT + PCL + VEGF grafts transplantation; Group E, VADT + PCL + EPCs grafts transplantation; and Group F, VADT + PCL + VEGF + EPCs grafts transplantation. In Groups A, B, and C, the tracheal grafts were used for orthotopic transplantation directly. In Group D, 100 μL of Matrigel containing 1 μg of VEGF was injected at the outer surface of the graft with a 1‐mL syringe after the graft anastomosis was completed. In Groups E and F, the tracheal grafts were placed horizontally (the same orientation as three‐point bending and compression) on a 24‐well plate and 1 mL of EPCs (1 × 10^6^/mL) suspension was added before 48 h of operation; after 24 h of incubation in the incubator, the culture medium was removed, the segments were rotated by 180° and EPCs were added to the other half of the luminal and outer surface, and the culture was incubated for another 24 h before the orthotopic transplantation. Furthermore, in Group F, 100 μL of Matrigel containing 1 μg of VEGF and 1 × 10^6^ of EPCs was injected at the outer surface of the graft after the graft anastomosis was completed.

#### 2.9.2 Scanning electron microscopy

To evaluate the adhesion of EPCs to the luminal and external surfaces of the grafts after in vitro cell seeding, one graft each from Group D, Group E, and Group F was fixed in 2.5% (v/v) glutaraldehyde for 24 h. After rinsing in PBS, the specimens were dehydrated through an ethanol gradient, dried at the critical point, sputter plated with gold, and observed by SEM (S‐4800, Hitachi High‐Technologies Corporation, Tokyo, Japan).

#### 2.9.3 Anesthesia and perioperative management

Anesthesia was induced using isoflurane (5%, RED, Shenzhen, China) mask inhalation for 2 min. Anesthesia was maintained with an ethyl carbamate (20%, 2 mL/kg; Aladdin, Shanghai, China) intravenous injection combined with isoflurane (2%) inhalation and spontaneous breathing was maintained until the skin was sutured. Standard surgical procedures were performed for tracheal transplantation. The skin of the neck was sterilized three times with medical iodophor, and the fascia and muscles were incised in the anterior midline of the neck to fully expose the cervical trachea. A 1‐cm long autologous tracheal segment was excised 2 cm distal to the cricoid cartilage and replaced with the transplanted trachea. A 4–0 absorbable suture (VCP771D, Ethicon, NJ, USA) was used for the continuous suturing of the anastomosis. Antibiotics (benzylpenicillin potassium for injection, 50,000 U/kg intramuscular for BID; Mingyue Veterinary Medicine Co., Suzhou, China) were administered intramuscularly for 1 week postoperatively. None of the subject groups received immunosuppressive therapy.

#### 2.9.4 Postoperative specimen testing

The survival of experimental animal subjects was dynamically observed, their clinical symptoms of inflammation or rejection were checked, and their general health was assessed. At 4, 8, and 10 weeks after the surgery, one surviving animal was randomly selected from each group to be euthanized, and bronchoscopy (MBC‐5, Aohua, Shanghai, China) was performed to observe anastomotic and airway patency. The grafts were then obtained using standard surgery. The samples in each group were fixed in 4% paraformaldehyde for 24 h, and paraffin sections (6 μm) were prepared. H&E, MT, and SOG staining were performed to evaluate the changes in tissue structure. IF staining of anti‐alpha smooth muscle actin (α‐SMA, Servicebio, Wuhan, China), CD31 (GeneTex, Alton Pkwy Irvine, CA, USA), and vWF (Bioss, Beijing, China) were performed to assess microvascularization.

### 2.10 Statistical analysis

Statistical analysis was performed using one‐way analysis of variance (ANOVA) or a two‐tailed Student's *t*‐test using SPSS version 24.0 (SPSS, Chicago, IL, USA). Data histograms and survival were analyzed using GraphPad Prism (version 7.0, GraphPad Company, San Diego, USA). Flow cytometry was performed using FlowJo (version 10.0, BD Life Sciences, Ashland, USA). The microvessel density of immunofluorescent staining of postoperative specimens was performed using Image J (National Institutes of Health, USA). All data are reported as the mean ± standard deviation. Statistical significance was set at *p* < 0.05.

## 3 RESULTS

### 3.1 Preparation and characterization of VADT

**FIGURE 1:**
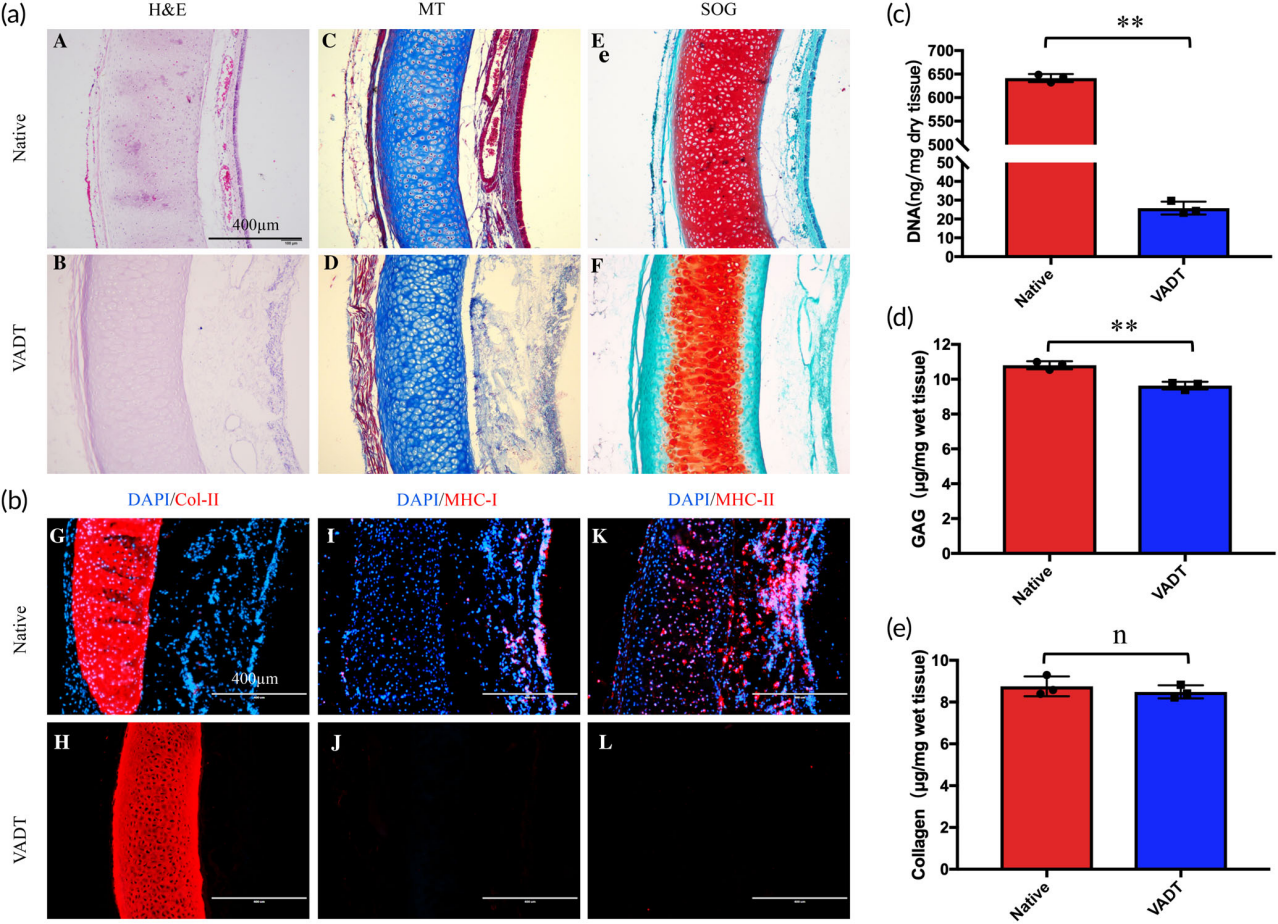
Histological and immunological evaluation of native and VADT grafts. (a) H&E staining (A, B), MT staining (C, D), and SOG staining (E, F) images of native and VADT grafts. (b) Immunofluorescence staining of DAPI/Col‐II (G, H), DAPI/MHC‐I (I, J), and DAPI/MHC‐II (K, L) expressed in the native trachea and VADT scaffolds. (c) DNA, (d) GAG, and (e) collagen quantitative analysis of native and VADT tissue. Scale bars = 400 μm. **p < 0.01; *n*, no statistical differences.

Histological staining using H&E, MT, and SOG was performed, and the epithelial, submucosal, and cartilaginous areas of the native and decellularized tracheal matrices were assessed microscopically for histological changes, as well as changes in the nuclei, fibers, and GAG content (Figure 1a). H&E staining showed that the nuclei were abundant in the epithelial layer, submucosa, and cartilage area of the native trachea, and were clearly removed in the VADT group, while the tissue structure remained intact (Figure 1a, A–B). MT staining showed abundant collagen and muscle fibers located in the cartilage and adventitia areas of the native trachea, respectively. After decellularization, collagen and muscle fibers were still fully expressed in the VADT group (Figure 1a, C–D).

Fluorescence microscopy showed that MHC‐I was primarily expressed in the mucosa and submucosa of the native trachea (Figure 1b, I), and MHC‐II was expressed abundantly not only in the mucosa and submucosa, but also in the cartilage area of the native trachea (Figure 1b, K). After decellularization, MHC‐I (Figure 1b, J), MHC‐II (Figure 1b, L), and DAPI expression almost completely disappeared in the VADT scaffold. DNA quantitative analysis shows that the DNA content in VADT scaffolds (25.80 ± 3.43 ng/mg) was significantly lower than in the native group (641.55 ± 8.55 ng/mg). Compared with that in the native group, only 4% of the DNA content remained in the VADT group (*p* < 0.01, Figure 1c). Three relatively stringent criteria have been proposed to confirm the non‐immunogenicity of decellularized tissue: (1) double‐stranded DNA content less than 50 ng/mg dry weight, (2) DNA fragments length less than 200 base pairs, and (3) no visible nuclear material by H&E or DAPI staining analysis.^37^, ^38^ Given these criteria, our results showed that VADT was cleared of any immunogenic material.

SOG staining showed that GAG was abundantly expressed in the cartilage of the native trachea. After decellularization, the expression of GAG was still evident in the VADT group (Figure 1a, E–F). GAG quantitative analysis showed that the GAG content in VADT scaffolds (9.64 ± 0.22 μg/mg) was significantly lower than in the native group (10.8 ± 0.23 μg/mg) (*p* < 0.01, Figure 1d). However, 89% of the GAG content was retained in the VADT group compared with that in the native group. IF staining showed that Col‐II was highly expressed in the cartilage area of both the native trachea and VADT scaffolds (Figure 1b, G–H). There was no difference in the total collagen content between the VADT group (8.49 ± 0.31 μg/mg) and the native group (8.76 ± 0.48 μg/mg) (*p* > 0.05, Figure 1e).

### 3.2 Fabrication and characterization of 3D hybrid scaffolds

Although the VADT scaffold retained an intact extracellular matrix structure while becoming non‐immunogenic, the longitudinal compression performance was significantly reduced. PCL is widely used in regenerative medicine, owing to its good mechanical properties and biocompatibility.^39^ To extract the respective advantages of natural and synthetic materials and further improve the biomechanical properties of VADT, 3D printed PCL stents were prepared, and hybrid tracheal grafts were constructed. The morphology of the 3D printed stents and VADT/PCL hybrid grafts are shown in Figure S1.

**FIGURE 2:**
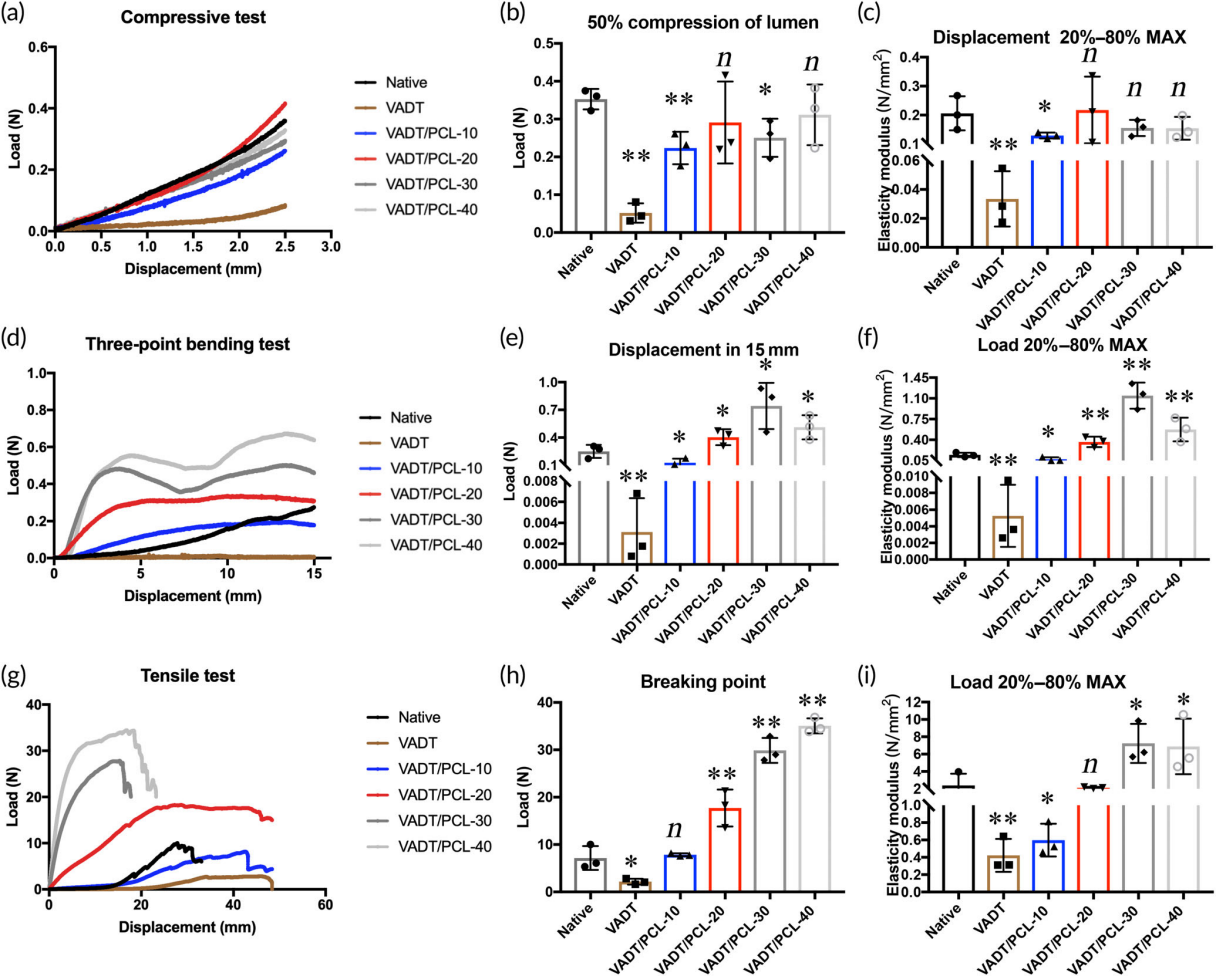
Biomechanical performance testing of native, VADT, VADT/PCL‐10, VADT/PCL‐20, VADT/PCL‐30, and VADT/PCL‐40 groups. In compressive tests, (a) the curve diagram of the load changes corresponding to the displacement of each group; (b) the load of 50% compression of lumen for each group; (c) the elastic modulus of 20%–80% maximum displacement for each group. In three‐point bending test; and (d) the curve diagram of the load changes corresponding to the displacement of each group; (e) the displacement load in 15 mm for each group; and (f) the elastic modulus of 20%–80% maximum load for each group. In tensile tests, (g) the curve diagram of the load changes corresponding to the displacement of each group; (h) the load of breaking test for each group; and (i) the elastic modulus of 20%–80% maximum load for each group. *p < 0.05; **p < 0.01; *n*, no statistical differences.

The curve diagrams of load changing in correspondence with the displacement of native, VADT, VADT/PCL‐10, VADT/PCL‐20, VADT/PCL‐30, and VADT/PCL‐40 group scaffolds in compressive tests are shown in Figure 2a. The load of 50% compression of lumen for the VADT/PCL‐20 (0.29 ± 0.11 N) and VADT/PCL‐40 (0.31 ± 0.08 N) scaffolds were comparable to that of the native group (0.35 ± 0.03 N) (*p* > 0.05), while the load of the VADT (0.05 ± 0.03 N), VADT/PCL‐10 (0.22 ± 0.04 N) and VADT/PCL‐30 (0.25 ± 0.05 N) scaffolds were significantly lower than that of the native tracheae (*p* < 0.05) (Figure 2b); the elastic modulus of between 20% and 80% max displacement for the VADT/PCL‐20 (0.22 ± 0.12 N), VADT/PCL‐30 (0.16 ± 0.03 N), and VADT/PCL‐40 (0.15 ± 0.04 N) scaffolds was comparable to that of the native group (0.21 ± 0.06 N) (*p* > 0.05), while the elastic modulus of the VADT (0.03 ± 0.02 N) and VADT/PCL‐10 (0.13 ± 0.01 N) scaffolds was significantly lower than that of the native tracheae (*p* < 0.05) (Figure 2c). The curve diagrams of the load changing in correspondence with the displacement of native, VADT, VADT/PCL‐10, VADT/PCL‐20, VADT/PCL‐30, and VADT/PCL‐40 group scaffolds in three‐point bending tests are shown in Figure 2d. The three‐point bending test showed that the load of displacement in 15 mm for the VADT/PCL‐20 (0.40 ± 0.09 N), VADT/PCL‐30 (0.74 ± 0.25 N) and VADT/PCL‐40 (0.51 ± 0.13 N) scaffolds was significantly higher than that of the native group (0.25 ± 0.07 N) (*p* < 0.05), while the load of the VADT (0.003 ± 0.003 N) and VADT/PCL‐10 (0.13 ± 0.05 N) scaffolds was significantly lower than that of the native tracheae (*p* < 0.05) (Figure 2e); the elastic modulus of between 20% and 80% max load for the VADT/PCL‐20 (0.37 ± 0.09 N), VADT/PCL‐30 (1.15 ± 0.22 N), and VADT/PCL‐40 (0.57 ± 0.20 N) scaffolds was significantly higher than that of the native group (0.15 ± 0.04 N) (*p* < 0.01), while the constant load of the VADT (0.005 ± 0.004 N) and VADT/PCL‐10 (0.07 ± 0.03 N) scaffolds was significantly lower than that of the native tracheae (*p* < 0.05) (Figure 2f). The curve diagrams of the constant load changing in correspondence with the displacement of native, VADT, VADT/PCL‐10, VADT/PCL‐20, VADT/PCL‐30, and VADT/PCL‐40 group scaffolds in tensile tests are shown in Figure 2g. The tensile test showed that the load at breaking point for the VADT/PCL‐20 (17.70 ± 3.90 N), VADT/PCL‐30 (29.88 ± 2.62 N), and VADT/PCL‐40 (35.07 ± 1.57 N) scaffolds was significantly higher than that for the native group (7.15 ± 2.52 N) (*p* < 0.01), while the load of the VADT (2.17 ± 0.61 N) scaffolds was significantly lower than that of the native tracheae (*p* < 0.05), and the VADT/PCL‐10 (7.85 ± 0.29 N) scaffolds were comparable to those of the native group (Figure 2h); the elastic modulus of between 20% and 80% max load for the VADT/PCL‐30 (7.25 ± 2.25 N) and VADT/PCL‐40 (6.89 ± 3.21 N) scaffolds was significantly higher than that of the native group (2.38 ± 1.37 N) (*p* < 0.05), while the load of the VADT (0.42 ± 0.19 N) and VADT/PCL‐10 (0.60 ± 0.19 N) scaffolds was significantly lower than that of the native tracheae (*p* < 0.05); the VADT/PCL‐20 (2.12 ± 0.10 N) scaffolds were comparable to that of the native group (Figure 2i).

In summary, the biomechanical properties of VADT/PCL‐20, VADT/PCL‐30, and VADT/PCL‐40 were slightly stronger than those of the native trachea in terms of compression, tension, and three‐point bending tests, while VADT and VADT/PCL‐10 were weaker than the native trachea (Table S1).

Figure S2A shows the result of the mass loss of VADT/PCL‐20 scaffolds in PBS. Statistical analysis revealed significant differences between the three scaffolds of weight loss for all time points. The weight loss of VADT/PCL‐20 scaffolds in 28 days was 13.38 ± 1.09%. Mechanical properties of VADT/PCL‐20 scaffolds during degradation in vitro are shown in Figure S2B. No significant changes in the elastic modulus value over 28 days during degradation were observed (*p* > 0.05).

### 3.3 Extraction and identification of EPCs

**FIGURE 3:**
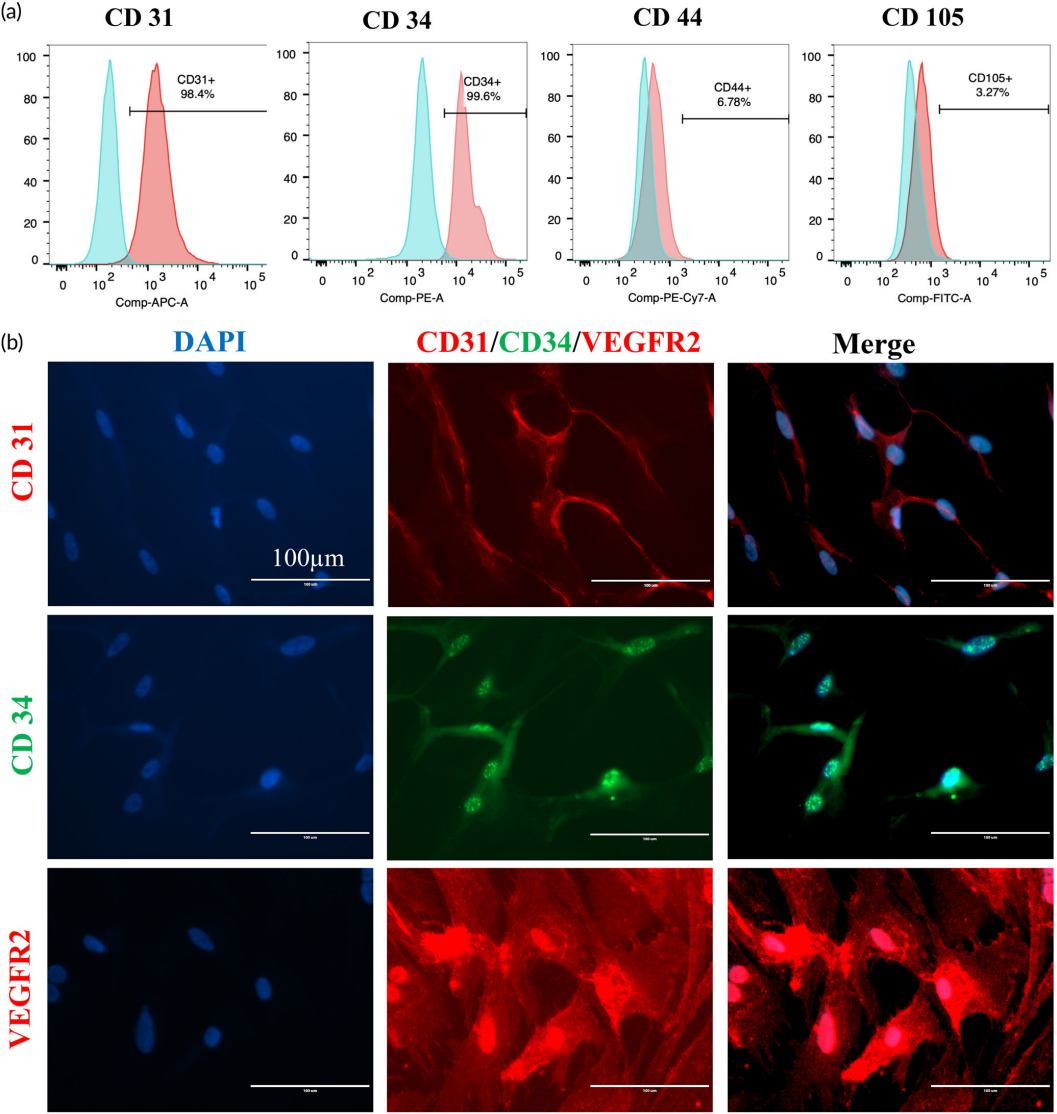
Extraction and identification of EPCs. (a) Flow cytometry analysis expressing CD31, CD34, CD44, and CD105 of EPCs. The blue histograms correspond to cells unstained with fluorescent antibodies; the red histograms correspond to cells stained with fluorescent antibodies. (b) Immunofluorescence staining reveals the expression of CD31, CD34, and VEGFR2 of EPCs. Scale bars = 100 μm.

In this study, the whole BM differential adherence method combined with an EGM‐2MV medium was used to isolate and culture the EPCs. Flow cytometry analysis showed that CD31 (positive rate 98.4%) and CD34 (positive rate 99.6%) were expressed at significantly high levels, whereas CD44 (positive rate 6.78%) and CD105 (positive rate 3.27%) were expressed at significantly low levels in the group of EPCs (Figure 3a). The IF staining showed that the CD31, CD34, and VEGFR2 were expressed significantly in these cells (Figure 3b). These experimental results comprehensively indicated that the extracted cells were EPCs.

### 3.4 Biocompatibility of 3D hybrid scaffolds

**FIGURE 4:**
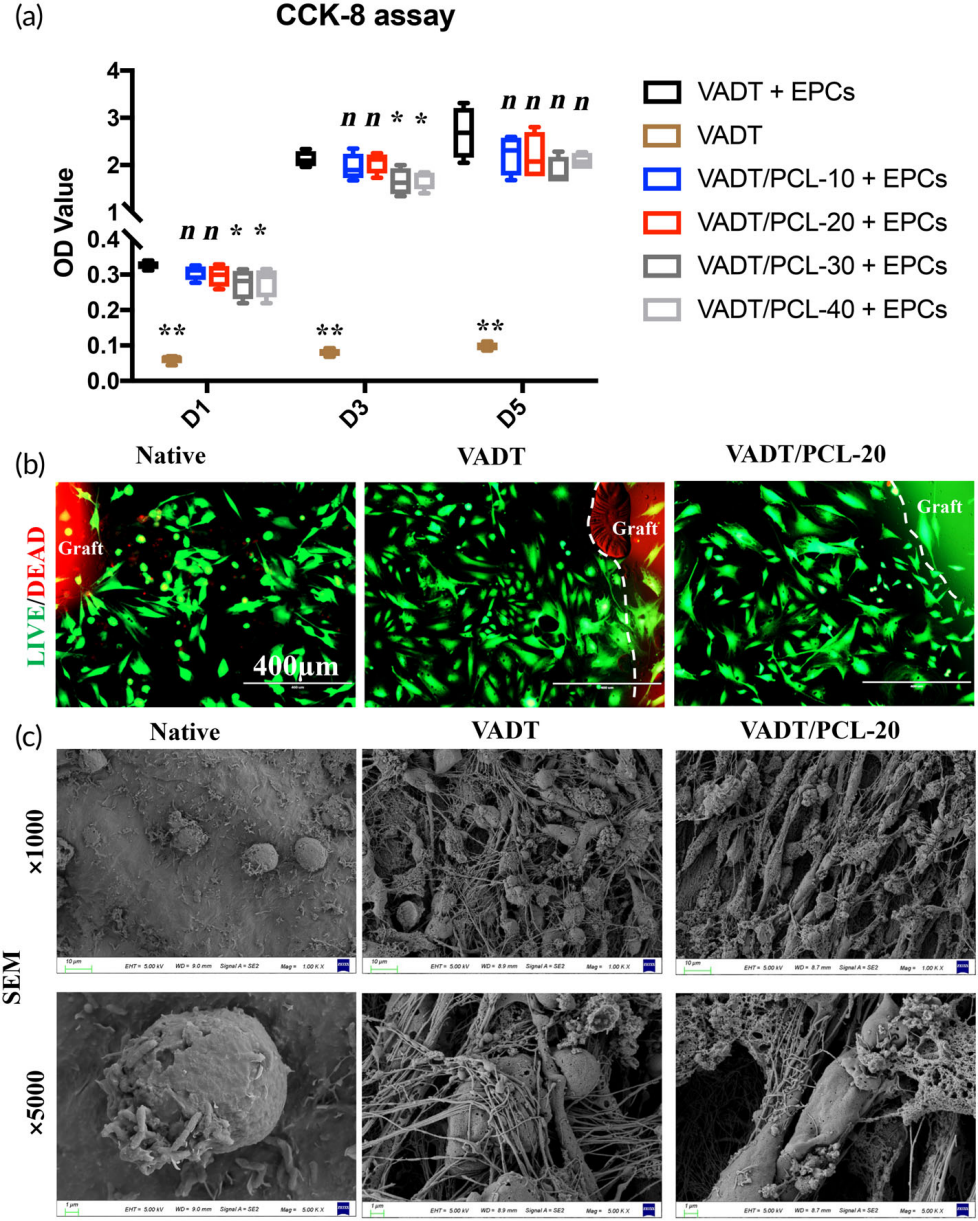
Biocompatibility of VADT, VADT/PCL‐10, VADT/PCL‐20, VADT/PCL‐30, and VADT/PCL‐40 scaffolds. (a) The CCK‐8 assays demonstrated the OD values of each group with or without seeding EPCs on Days 1, 3, and 5. (b) Live–dead cell experiments showed that few cells died around the graft in the native trachea group, while the cells around the graft in the VADT and the VADT/PCL‐20 scaffolds survived. Scale bars = 400 μm. (c) SEM with obvious cell adhesion growth on the surface of the graft in the native, VADT, and VADT/PCL‐20 scaffolds. Scale bars = 10 or 1 μm.

Excellent biocompatibility is a prerequisite for promoting cell adhesion and proliferation on the graft luminal surface. Therefore, in this study, EPCs were seeded on the luminal surface of the graft in vitro, and the biocompatibility of VADT and hybrid scaffolds was verified by CCK‐8 assays, live and dead cell experiments, and SEM. The CCK‐8 assays demonstrated that the OD values of VADT/PCL‐10 + EPCs and VADT/PCL‐20 + EPCs groups were not statistically different than those of the VADT group on Days 1, 3, and 5 (*p* > 0.05); those of VADT, VADT/PCL‐30 + EPCs, and VADT/PCL‐40 + EPCs groups were significantly lower than that of the VADT group on Days 1 and 3 (*p* < 0.05) (Figure 4a; Table S2). This indicated that the VADT/PCL‐10 and VADT/PCL‐20 groups of grafts were more suitable for cell adhesion and proliferation than the VADT/PCL‐30 and VADT/PCL‐40 groups. Live–dead cell experiments showed that the number of cells that died around the graft in the native trachea group owing to its strong immunogenicity was higher than that around the graft in the VADT and VADT/PCL‐20 groups, indicating better survival rate in the case of the latter (Figure 4b). SEM results showed cell adhesion on the luminal surface of the graft in the native, VADT, and VADT/PCL‐20 groups (Figure 4c).

### 3.5 In vivo angiogenic properties

**FIGURE 5:**
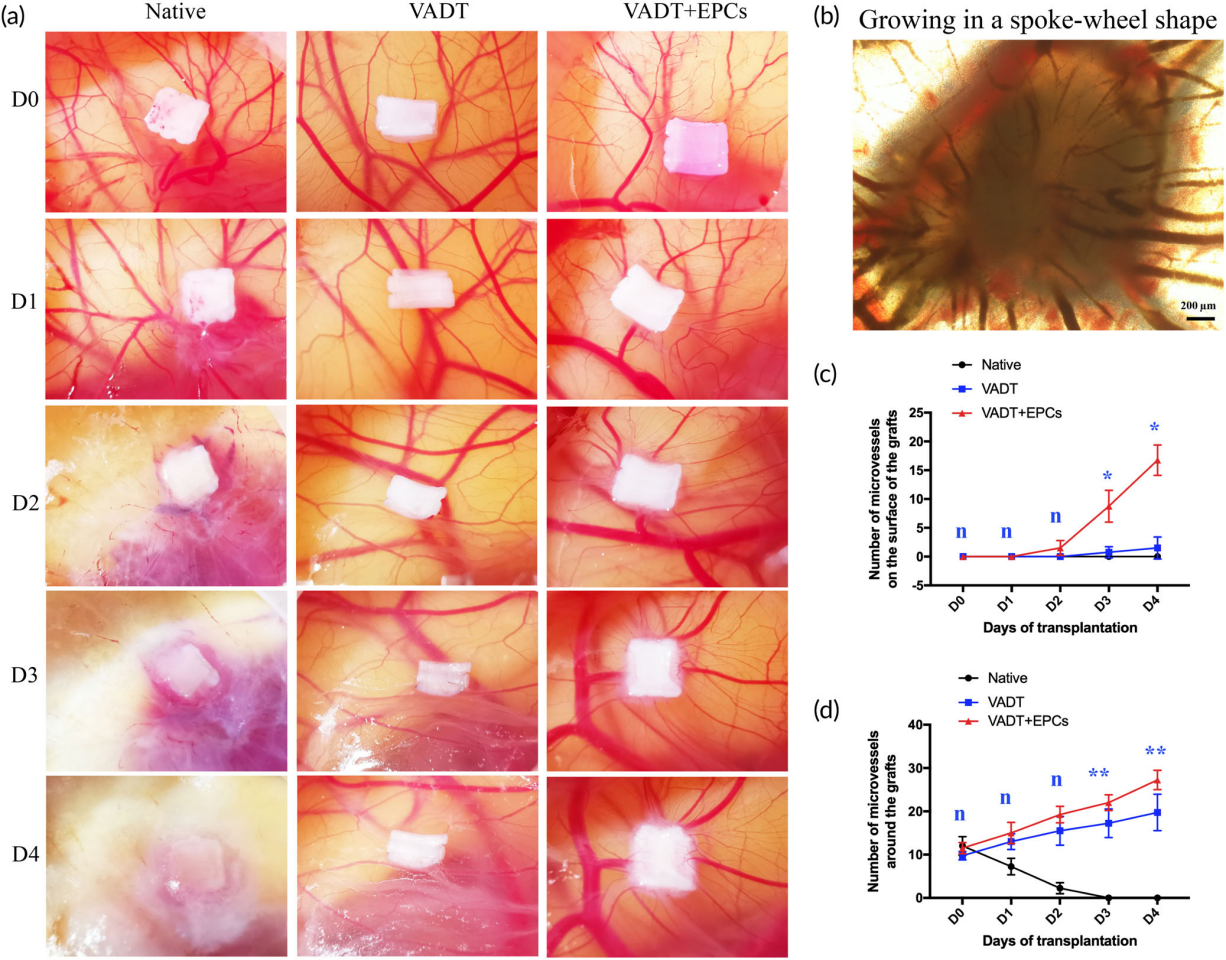
In vivo angiogenic properties of native, VADT, and VADT + EPCs scaffolds. (a) CAM shows the angiogenic properties around the grafts of native, VADT, and VADT + EPCs scaffolds on Days 0, 1, 2, 3, and 4 after transplantation. (b) Neovascularization growth around the graft of VADT+EPCs scaffolds (spoke‐wheel shape). Scale bars = 200 μm. (c) Amount of microvascular network growth around the graft higher in the VADT + EPCs group than in the VADT group after Day 3. (d) Clear microvascularization on the graft surface in the VADT + EPCs group. *p < 0.05; **p < 0.01; *n*, no statistical differences.

The microvascularization of grafts in vivo is especially critical for their proper functioning and the long‐term survival of transplant receptors. CAM was used to evaluate the effect of microvascularization of the VADT scaffolds promoted by EPC seeding in vivo (Figure 5). The chick embryos died on the second day after native tissue implantation, owing to the strong immunogenicity of the native trachea. Two days after the implantation of the matrix in the VADT group, microvascular angiogenesis gradually appeared around the graft, growing in a spoke‐wheel shape. This indicates that VADT can induce angiogenesis in vivo and is non‐immunogenic. Vascular growth around the grafts in the VADT + EPC group was similar to that in the VADT group in the first 2 days after implantation. However, the number of microvascular network growths around the graft was higher in the VADT + EPCs group than in the VADT group after the third day. More importantly, there was no microvascularization on the graft surface in the pure VADT group; however, there was clear microvascularization on the graft surface in the VADT + EPCs group. This indicates that EPCs effectively promoted the microvascularization of VADT in vivo.

**FIGURE 6:**
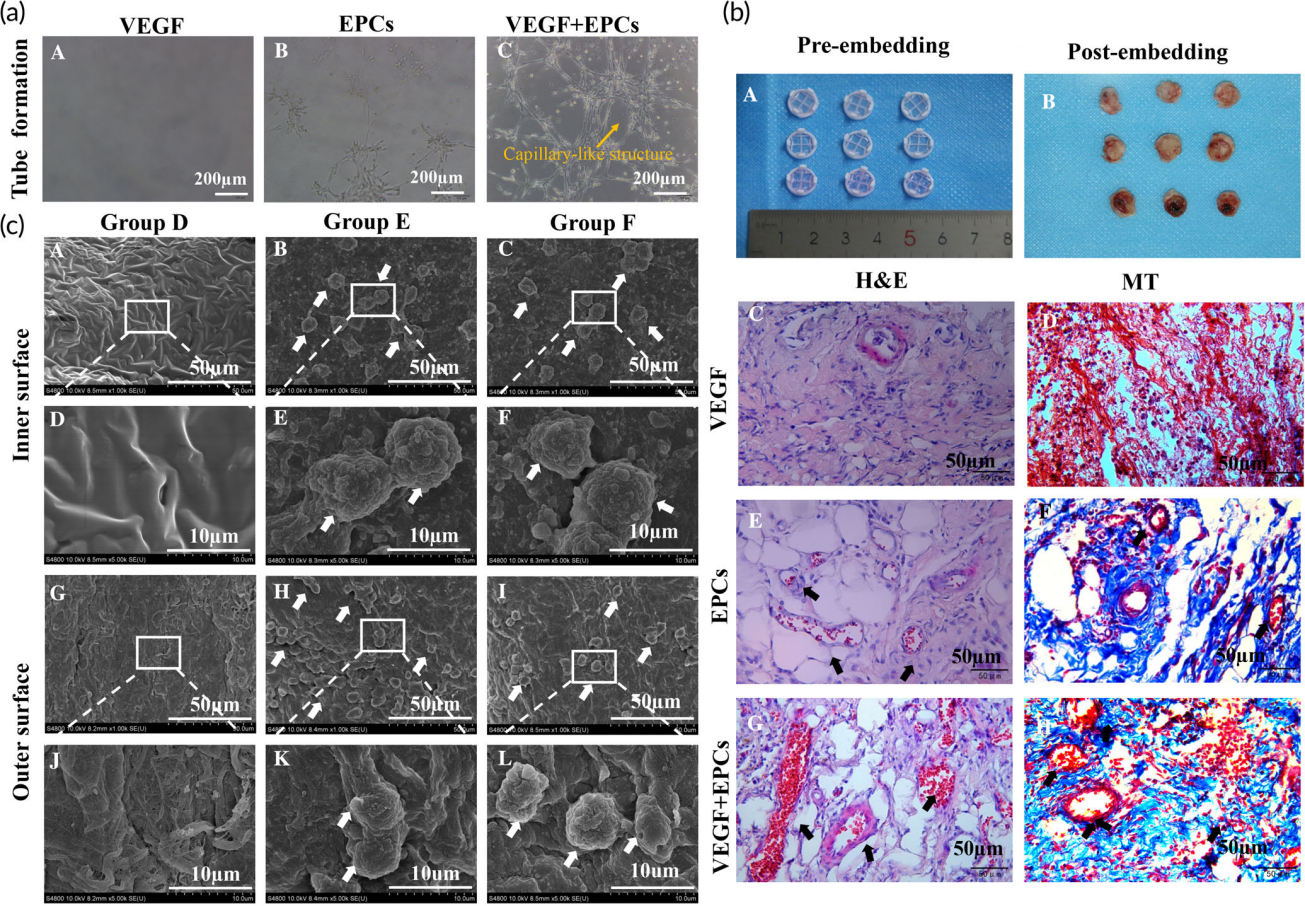
Vascular‐like structure formation properties and adhesion properties on graft surfaces of EPCs. (a) In vitro tube formation of EPCs on the surface of Matrigel. Scale bars = 200 μm. (C) Capillary‐like structures significantly enhanced in combination with VEGF and EPC group compared with the EPC‐alone group. (b) Subcutaneous embedding of Matrigel mixed with VEGF alone, EPC alone, and both VEGF and EPC for 1 week in rats. Scale bars = 50 μm. (A, B) Gross images; (C, E, G) H&E stains, and (D, F, H) MT stains. Black arrows indicate microvascular structures. (c) EPC adhesion on the luminal and outer surfaces of the graft after seeding in vitro. White arrows indicate adherent cells. Scale bars = 50 or 10 μm.

In vitro tube formation experiments demonstrated that EPCs alone had a slightly poorer growth performance in vascular‐like structures on the surface of Matrigel (Figure 6a, B); in combination with VEGF, the growth performance of EPCs in vascular‐like structures was significantly enhanced (Figure 6a, C). The subcutaneous embedding of Matrigel mixed with VEGF alone, EPCs alone, and both VEGF and EPCs for 1 week showed that the in vivo microvascular formation effect of Matrigel mixed with VEGF alone or EPCs alone was less than that when they were combined, indicating that the rational use of VEGF can further enhance the in vivo microvascular formation of EPCs (Figure 6b).

### 3.6 Orthotopic transplantation studies

**FIGURE 7:**
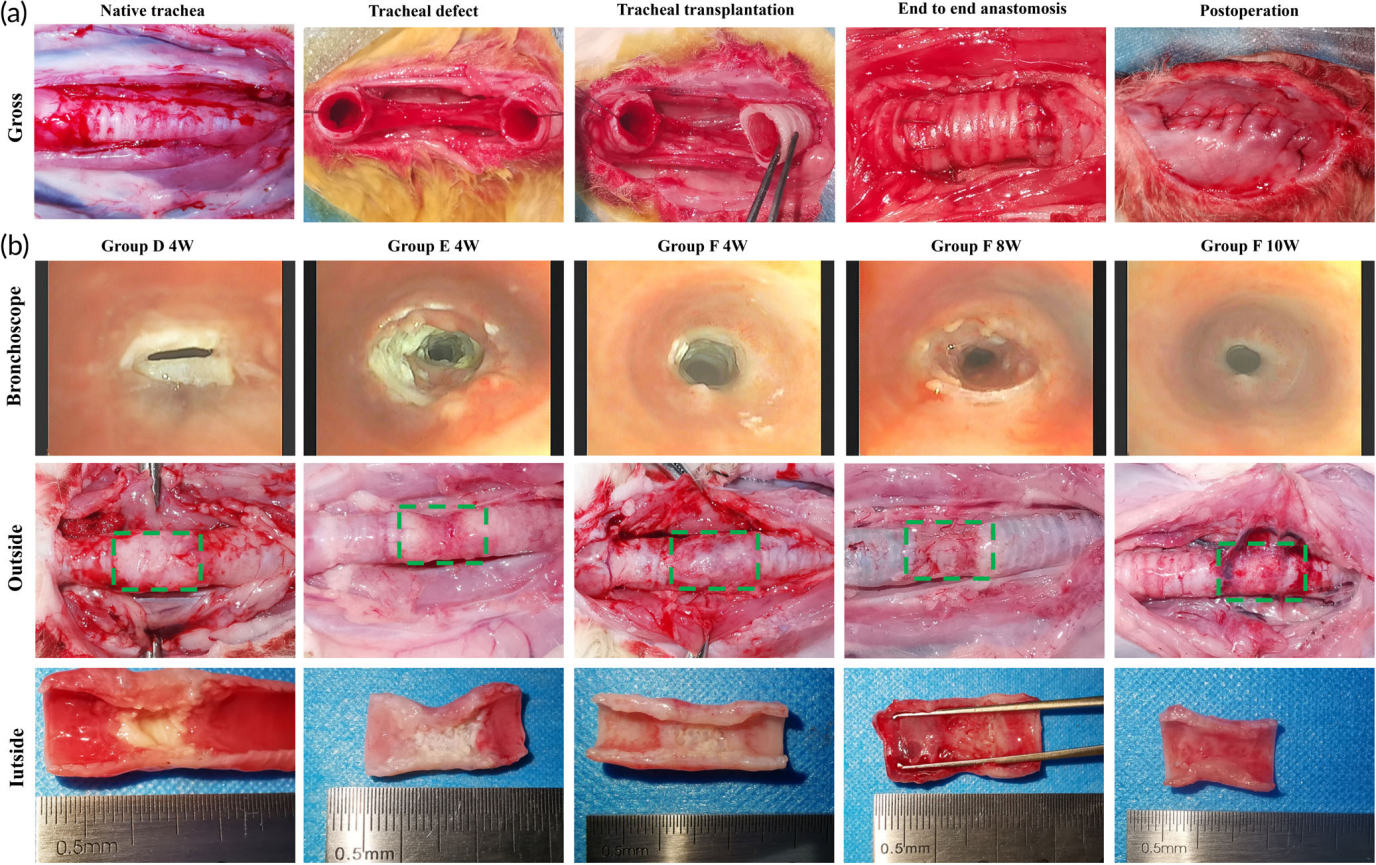
(a) Tracheal transplantation in a rabbit model system to evaluate the preclinical utility of biomimetic in situ tracheal microvascularization for long‐segment tracheal reconstruction in one step. (b) Bronchoscopy and macroscopic observations. Group D, VADT + PCL + VEGF grafts transplantation; Group E, VADT + PCL + EPC grafts transplantation; and Group F, VADT + PCL + VEGF + EPC grafts transplantation.

We subsequently performed a tracheal transplantation in a rabbit model system to further evaluate the preclinical utility of biomimetic in situ tracheal microvascularization for segment tracheal reconstruction in one‐step. SEM showed significant cell adhesion on the luminal and external surfaces of the grafts in Groups E and F after preoperative cell seeding in vitro (Figure 6c). Without ectopic embedding for microvascularization, the grafts were used to repair a segmental tracheal defect using end‐to‐end anastomosis directly (Figure 7a). None of the rabbits in Groups A, B, and C survived for 4 weeks, owing to anastomotic stenosis, tracheomalacia, or asphyxia due to phlegm blockage. In Groups D and E, the 4‐week survival rate was only 20%, and the main causes of death were anastomotic stenosis, graft necrosis, and phlegm blockage asphyxiation. In contrast, 80% of the rabbits survived for 4 weeks with no evidence of respiratory distress in Group F (Table 1). The surviving rabbits were euthanized for postoperative evaluation after 4, 8, or 10 weeks. Bronchoscopy and macroscopic observations showed that the inner wall of the graft in Groups D and E at 4 weeks was pale in color and lacked vascularization. However, in Group F, the inner and outer walls of the graft were ruddy in color at 4, 8, and 10 weeks, and the lumen was unobstructed, which suggests microvascularization (Figure 7b).

**TABLE 1:** Survival analysis and main cause of death of experimental animals in each group.

Group	Survival for 4 weeks	Cause of death
Group A	0/0 (0%)	Choking due to phlegm (1/5 2 days), stenosis (1/5 6 days), and necrosis and infection (2/5 7 days, 1/5 9 days)
Group B	0/0 (0%)	Choking due to phlegm (1/5 7 days), stenosis (2/5 10 days), and airway collapse (1/5 6 days, 1/5 11 days)
Group C	0/0 (0%)	Choking due to phlegm (1/5 12 days, 1/5 17 days), necrosis and infection (1/5 21 days, 1/5 25 days), and stenosis (1/5 26 days)
Group D	1/5 (20%)	Choking due to phlegm (1/5 12 days, 1/5 17 days), necrosis (1/5 21 days), stenosis (1/5 25 days), and euthanasia (1/5 4 weeks)
Group E	1/5 (20%)	Choking due to phlegm (1/5 11 days, 1/5 18 days, 1/5 22 days), stenosis (1/5 27 days), and euthanasia (1/5 4 weeks)
Group F	4/5 (80%)	Choking due to phlegm (1/5 22 days, 1/5 35 days), euthanasia (1/5 4 weeks, 1/5 8 weeks), and stenosis (1/5 10 weeks)

**FIGURE 8:**
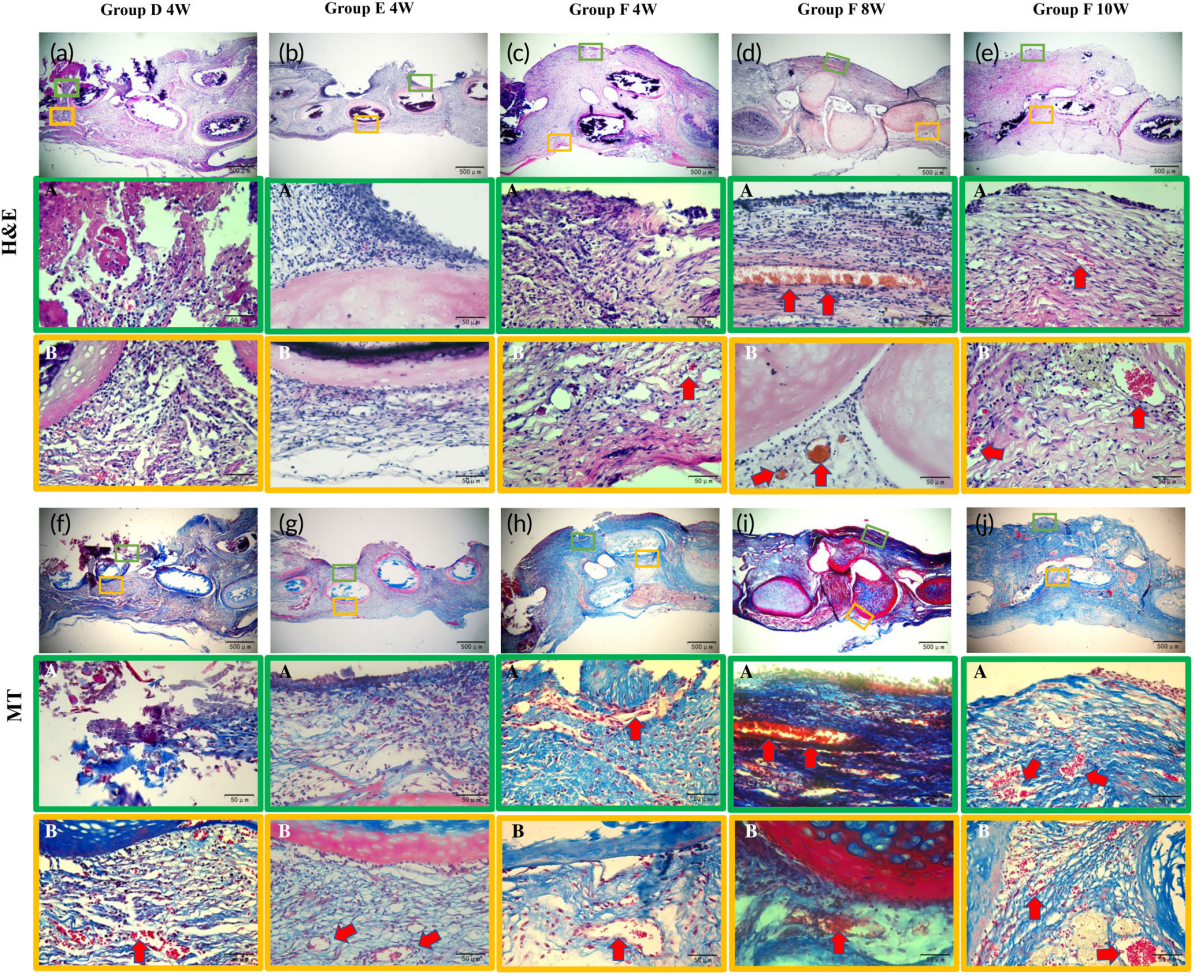
(a–e) H&E and (f–j) MT staining of the histology of grafts for recipients surviving more than 4 weeks. Scale bars = 500 μm. (B) Small yellow boxes indicate the mucosal layer, shown at higher magnifications. (A) Small green boxes indicate the adventitial area, shown at higher magnifications. Scale bars = 50 μm.

**FIGURE 9:**
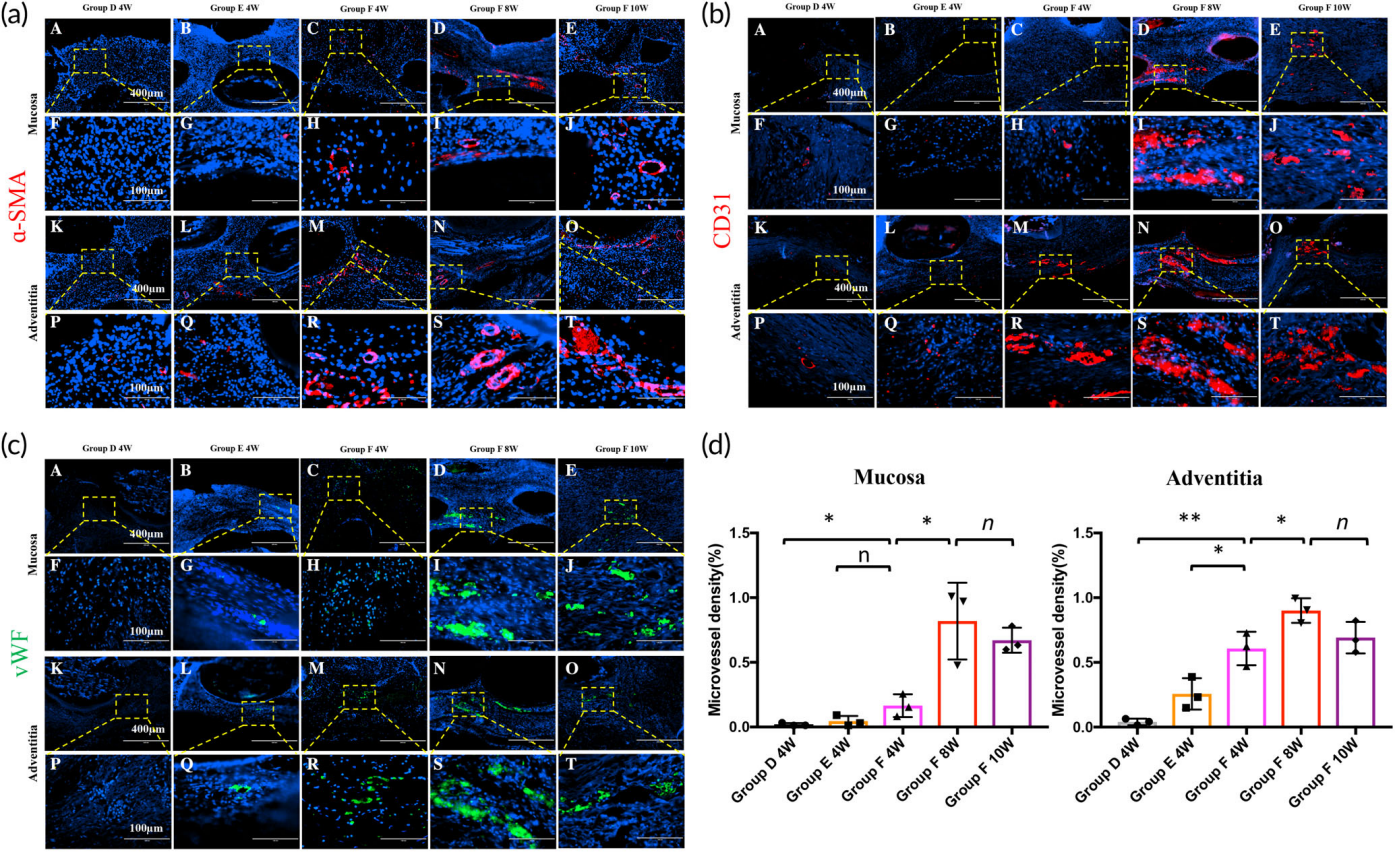
Immunofluorescence showing expression of (a) α‐SMA, (b) CD31, and (c) vWF of grafts for the recipients in Groups D, E, and F surviving more than 4 weeks. Clear expression of α‐SMA, CD31, and vWF in the mucosal and adventitial regions of the grafts in Group F. (d) Microvessel density of grafts supplemented to indirectly quantify microvascular formation. (A–E, K–O) Scale bars = 400 μm; (F–J, P–T) scale bars = 100 μm. *p < 0.05; **p < 0.01; *n*, no statistical differences.

The results of H&E and MT staining showed that visible microvascular structures were observed in the adventitial area of tracheal grafts in Groups D, E, and F at 4 weeks. However, incomplete mucosal tissue structure and no microvascular structure were observed in the tracheal grafts of Groups D and E, respectively, at 4 weeks. Visible microvascular structures were observed both in the mucosal layer and the adventitial area with no clear inflammatory cell infiltration of tracheal grafts Group F at 4 weeks. Furthermore, microvascularization of the tracheal graft became more pronounced at 8 or 10 weeks after transplantation (Figure 8). IF showed that the expression of α‐SMA, CD 31, and vWF were clear in the mucosal and adventitial regions of the grafts in Group F. However, the α‐SMA, CD 31, and vWF expression was lower in Groups D and E, which demonstrated that EPC combined with VEGF can effectively promote microvascularization in VADT/PCL grafts in situ (Figure 9a–c). To further quantify the effect of microvessel formation, the microvessel density was determined and was found to be the most significant both in mucosa and adventitia of grafts in Group F than in Group D or E 4 weeks after transplantation. A further increase in microvessel density of grafts at 8 weeks compared with that at 4 weeks of postoperation in Group F was observed; however, no further increase was recorded at 10 weeks (Figure 9d).

## 4 DISCUSSION

Although tissue‐engineering technology has provided considerable development opportunities for tracheal reconstruction surgery, it also faces many challenges, such as the preparation of low‐immunogenic tracheal grafts, maintaining good biomechanical properties of the grafts, and rapid microvascularization of the grafts. These are the key challenges that determine the success of tracheal‐replacement therapy and the long‐term survival of the recipients. In this study, we developed a novel hybrid scaffold with extremely low immunogenicity, suitable biomechanical properties, and good biocompatibility for tissue‐engineered tracheal transplantation. Furthermore, we developed a new method for the microvascularization of tracheal grafts in one step, which directly promoted the microvascular network formation of the graft in situ. Rapid vascularization is crucial for cell proliferation, differentiation, and migration that effectively promote graft epithelialization, functionalization, and the long‐term survival of transplant recipients.^30^

In the preparation of a full biomimetic tracheal graft, it is important to select a suitable tracheal scaffold that has extremely low immunogenicity, a complete extracellular matrix structure, no toxic side effects, and is suitable for cell adhesion and proliferation. It has been confirmed that a natural decellularized tracheal extracellular matrix (dtECM) that meets the above requirements can be effectively obtained using decellularization.^20^, ^35^, ^36^ It has been demonstrated that dtECM supports neo‐epithelialization, endothelialization, and chondrocyte viability,^40^ and it may serve as a promising biomaterial for tracheal reconstitution.^41^ Although natural dtECM meets the requirements of most idealized tracheal grafts, the critical limitation is that the biomechanical properties of the scaffold are considerably reduced, resulting in serious complications, such as the softening and stenosis of the grafts in the early stage after orthotopic transplantation.^14^, ^42^ Pure synthetic tracheal grafts have favorable biomechanical properties; however, they exhibit numerous graft‐related complications, including obstructive granulation tissue and anastomotic leaks, lack of vascularization, epithelial lining, or fusion into surrounding tissues.^43^ Therefore, PCL stents that were matched with the native trachea were prepared using 3D printing technology. Hybrid tracheal grafts were constructed using VADT and PCL stent; the advantages of both natural and synthetic materials were utilized to further optimize the performance of the bionic tracheal grafts. Sun and colleagues^44^ demonstrated that PCL remained structurally intact in rats during 2 years of in vivo implantation and broke into low molecular weight pieces at the end of 30 months. Radiation tracer tests confirm that the material does not accumulate in tissues and can be completely excreted from the body. Dias and colleagues^45^ demonstrated that PCL degrades more slowly in vivo than in vitro and does not cause rejection in vivo. Therefore, the graft degradation in vivo was not performed in this research. In the following studies, tritium‐labeled hybrid scaffolds will be implanted into rabbits and tested for radioactive tracers in plasma, feces, and urine to study their absorption and excretion. Notably, the decellularized implants lack an epithelial layer; hence, they may not be suited to resolve complications associated with the elimination/clearance of mucus, airborne particulates, and bacteria. Therefore, promoting rapid epithelial cell migration from the host trachea growth on the graft luminal of the primary trachea is extremely important.

A critical challenge for the orthotopic transplantation of a tissue‐engineered trachea is the construction of the vascularization of the graft to provide nourishment to seed cells and promote the functionalization of neonatal tissues. Without effective microvascularization, cells in a biological microenvironment cannot acquire sufficient nutrients and oxygen or remove metabolic waste, which eventually leads to cell death and graft necrosis.^46^ Because the tracheal blood supply is primarily sourced from the inferior thyroid artery, superior bronchial artery, esophagotracheal microvascular network, and a lack of arteriovenous vessels for anastomosis, microvascularization of the tracheal graft using vascularized seed cells is particularly important. EPCs are superior to vascular‐derived ECs in forming vascular networks, and the vascular structures formed in vitro and in vivo possess permeability values similar to those of vascular‐derived ECs.^47^ Moreover, EPCs can be non‐invasively isolated from peripheral blood, BM, and umbilical cord blood, as well as from human induced pluripotent stem cells, avoiding trauma and immunogenicity issues.^48^, ^49^, ^50^, ^51^, ^52^ In this study, we isolated and cultured EPCs as microvascular seed cells using a differential adherence method. Flow cytometry analysis showed that CD31 (positive rate 98.4%) and CD34 (positive rate 99.6%) were significantly overexpressed, while CD44 (positive rate 6.78%) and CD105 (positive rate 3.27%) were underexpressed (Figure 3a). Furthermore, IF staining showed that the CD31, CD34, and VEGFR2 were significantly expressed in these cells (Figure 3b). Therefore, we identified the extracted cells as EPCs. In vivo experiments using a CAM confirmed clear microvascular formation both on the surface and around the matrix in the VADT + EPC group (Figure 5). This shows that EPCs can induce VADT angiogenesis in vivo without rejection.

Matrigel is the most frequently used material in determining angiogenesis by mimicking the physiological cell substances in vitro and in vivo.^53^ When VEGF is directly applied to scaffolds for angiogenesis, proper angiogenesis cannot occur due to the rapid diffusion of VEGF. Therefore, Matrigel could be used as a gel coating to maintain the VEGF concentration gradient and to control its release.^54^ In this study, in vitro tube‐forming assays demonstrated that the VEGF‐added group had more significant capillary‐like tubes of EPCs in Matrigel than the non‐VEGF added group. In vivo experiments demonstrated that Matrigel enriched with VEGF and EPCs exhibit significant angiogenesis after 1 week of embedding. However, Matrigel‐based organoid systems are best suited for laboratory investigations and not for clinical transplantation.^55^ Further validation of the reliability and reproducibility of composite hydrogels for organoid cultures and transplantations is essential for tissue replacement therapy and pharmaceutical applications in the coming years, although many technical challenges remain to be overcome; hence, selecting the appropriate scaffold and method of microvascularization is particularly important.

Many studies have attempted to promote tissue‐engineered tracheal revascularization, but there is still no consensus on how to construct microvascular grafts reasonably and effectively. Currently, there are three main strategies for vascularized construction of tracheal grafts: (1) heterotopic wrapping method, in which orthotopic transplantation is performed after the microvascularization of the grafts is completed in ectopic embedding in muscle flaps,^23^, ^56^, ^57^, ^58^, ^59^ forearm fascia,^6^, ^60^ or omentum^61^, ^62^ over several weeks; (2) promotion of angiogenesis using decellularized trachea that retains vascular growth factors^15^, ^16^; (3) 3D printing of microvascularized grafts using bioinks mixed with ECs and vascular growth factors.^63^ Although the above methods play a certain role in the microvascularization of tracheal grafts, they still have the following shortcomings: the ectopic wrapping method may require a secondary surgery, which is more traumatic; the decellularized trachea can easily soften and collapse after surgery, which requires repeated implantation of endotracheal stents; and the 3D bioprinted microvascularized tissue has weak biomechanical properties that cannot match the performance requirements of long‐segment tracheal grafts.^64^ However, the current study innovatively used a one‐step method to construct microvascularized tracheal grafts by preparing a VADT/PCL hybrid tracheal graft with good angiogenesis‐inducing and biomechanical properties. Tissue‐engineered grafts are avascular when implanted in vivo and need to induce vascular ingrowth to facilitate graft survival. To rapidly guide sprouting of new vessels and their migration into the graft, it is desirable for the graft matrix to present a natural biomimetic microenvironment that is favorable for angiogenesis. This can be achieved by employing decellularized ECM enriched in morphogens by suitable progenitor cell lines.^65^ To accelerate tracheal graft microvascularization in orthotopic transplantation directly, EPCs were seeded on the surface of the graft before the operation, and Matrigel containing VEGF and EPCs was injected at the outer surface of the graft after the anastomosis was completed. The results indicate that the EPCs combined with VEGF can effectively improve microvascularization during orthotopic transplantation of segmental VADT/PCL bionic trachea, thereby effectively improving the survival of transplanted animals. A 10‐week follow‐up may not be sufficient to account for all the potential mortalities, such as secretions/airway hygiene, stricture/collapse, and migration. However, this study aimed to dynamically observe the effect of microvascularization constructs in the short term after tracheal transplantation; significant microvascular outcomes were observed at 8 weeks postoperatively, with no further increase after 10 weeks. Therefore, the time node of 10 weeks was chosen. A long‐term (more than 6 months) survival outcome after tubular tracheal transplantation needs to be explored in the future.

The microvascular density of the grafts in this study was analyzed via analysis of thin section staining (e.g., CD31, ɑ‐SMA, and vWF). Unfortunately, “vascular staining” does not always correlate to physiologic perfusion. Providing another barometer of vascular quantification/perfusion, such as in situ laser Doppler or thermography of the engineered trachea, would be highly beneficial. A multitude of other methods could also be used, such as lectin/Evans Dye perfusion followed by light sheet microscopy. Hwang and colleagues monitored the time‐dependent changes of microvasculature using the optical‐resolution photoacoustic microscopy (OR‐PAM) system to investigate the efficacy of angiogenesis in vivo.^66^

In this study, single‐sex female New Zealand rabbits were chosen as transplant recipients for the following reasons: (1) mixing male and female rabbits can lead to unwanted pregnancies in females, which can create ethical problems and interfere with the results; (2) using a single sex can control for variable between different groups and prevent errors in the results due to the different physical conditions between male and female animals; (3) fighting is more common among male rabbits than among females, so selecting female rabbits can effectively reduce the possibility of unnecessary damage caused by fighting. The study design has been approved by the ethics committee and will not impact the translation of the results.

Although several efforts have been made to promote the clinical application of tissue‐engineered tracheas, there are still some limitations in this field that need to be urgently addressed. (1) The hybrid 3D tracheal graft we constructed has good biocompatibility as well as good angiogenesis‐inducing and biomechanical properties; however, its in vivo degradation and regeneration properties need further long‐term observation. (2) EPCs were not labeled; hence, there is no straightforward evidence that they contributed to microvascularization. Future research will focus on applying immunotracer techniques and dynamically observing microvascular formation. (3) Graft granulation tissue hyperplasia, lumen stenosis, and necrosis were the main causes of death in the experimental animals after tracheal transplantation. Effectively controlling anastomotic scar stenosis and promoting graft survival will be an additional focus of future research.

## 5 CONCLUSIONS

We developed a 3D printing macro‐PCL/VADT hybrid tracheal graft based on BM‐EPC seeding that supports vascularization in vivo. The hybrid tracheal graft has extremely low immunogenicity, good biocompatibility, angiogenesis‐inducing properties, and biomechanical properties that match those of the native trachea. Furthermore, EPCs were isolated and purified as vascularized seed cells using the differential adhesion method, which further promoted microvascularization in VADT in vivo. Subsequently, the segment hybrid tracheal graft was subjected to one‐step in situ replacement therapy in vivo. Additionally, EPCs were implanted preoperatively and intraoperatively combined with VEGF management, which rapidly promoted graft microvascularization. Therefore, this study demonstrated that the 3D printed macro‐PCL/VADT hybrid tracheal graft based on BM‐EPCs and VEGF management can be expected to provide a promising strategy for the rapid vascularization and clinical transplantation of tissue‐engineered tracheas.

## AUTHOR CONTRIBUTIONS

**Fei Sun:** Conceptualization (equal); data curation (equal); formal analysis (equal); investigation (equal); methodology (equal); visualization (equal); writing – original draft (equal); writing – review and editing (equal). **Zhiming Shen:** Investigation (equal); methodology (equal). **Boyou Zhang:** Conceptualization (supporting). **Yi Lu:** Investigation (supporting). **Yibo Shan:** Data curation (supporting). **Qiang Wu:** Resources (supporting). **Lei Yuan:** Methodology (supporting). **Jianwei Zhu:** Project administration (supporting). **Shu Pan:** Software (supporting). **Zhihao Wang:** Methodology (supporting). **Cong Wu:** Visualization (supporting). **Wenlong Yang:** Software (supporting). **Guozhong Zhang:** Methodology (supporting). **Xiangyu Xu:** Data curation (supporting). **Hongcan Shi:** Funding acquisition (lead); project administration (lead); supervision (lead).

## CONFLICT OF INTEREST STATEMENT

The authors declare no conflicts of interest.

### PEER REVIEW

The peer review history for this article is available at https://www.webofscience.com/api/gateway/wos/peer-review/10.1002/btm2.10534.

## Supporting information


**Figure S1.** Morphology of 3D printed stents and VADT/PCL hybrid grafts. (A) Macroscopic images of 3D printed (a) PCL‐10, (b) PCL‐20, (c) PCL‐30, and (d) PCL‐40 stents. (B) SEM images of the 3D printed (e) PCL‐10, (f) PCL‐20, (g) PCL‐30, and (h) PCL‐40 stents. (C) Macroscopic images of the (i) native trachea, (j) VADT, (k) assembly process of VADT/PCL hybrid grafts, and (l) different group of grafts (from left to right; native, VADT, VADT/PCL‐10, VADT/PCL‐20, VADT/PCL‐30, and VADT/PCL‐40 tracheal grafts). (D) Macroscopic images of (m) compressive, (n) three‐point bending, and (o) tensile tests.

**Figure S2.** Physical characterization of the VADT/PCL‐20 scaffolds. (A) Degradation characterization of the scaffolds (*n* = 3, *p* < 0.05). (B) Tensile mechanical properties of the scaffolds during degradation (*n* = 3, *p* > 0.05).

**Figure S3.** SOG staining showing the histology of grafts for recipients surviving more than 4 weeks. (F–J) Cartilage area of native tracheae. Scale bars = 50 μm. (K–O) Cartilage area of grafts. (A–E) Scale bars = 500 μm.

**Data S1.** Supporting Information.

## Data Availability

All data needed to evaluate the conclusions in the paper are present in the paper and/or the Supplementary Materials. Additional data related to this paper may be requested from the authors.
