# Supplementary figures and images for: Biomimetic in situ tracheal microvascularization for segmental tracheal reconstruction in one‐step
Source: Bioeng Transl Med. 2023 May 3;8(4):e10534. doi: 10.1002/btm2.10534 (PMC10354772; doi:10.1002/btm2.10534)

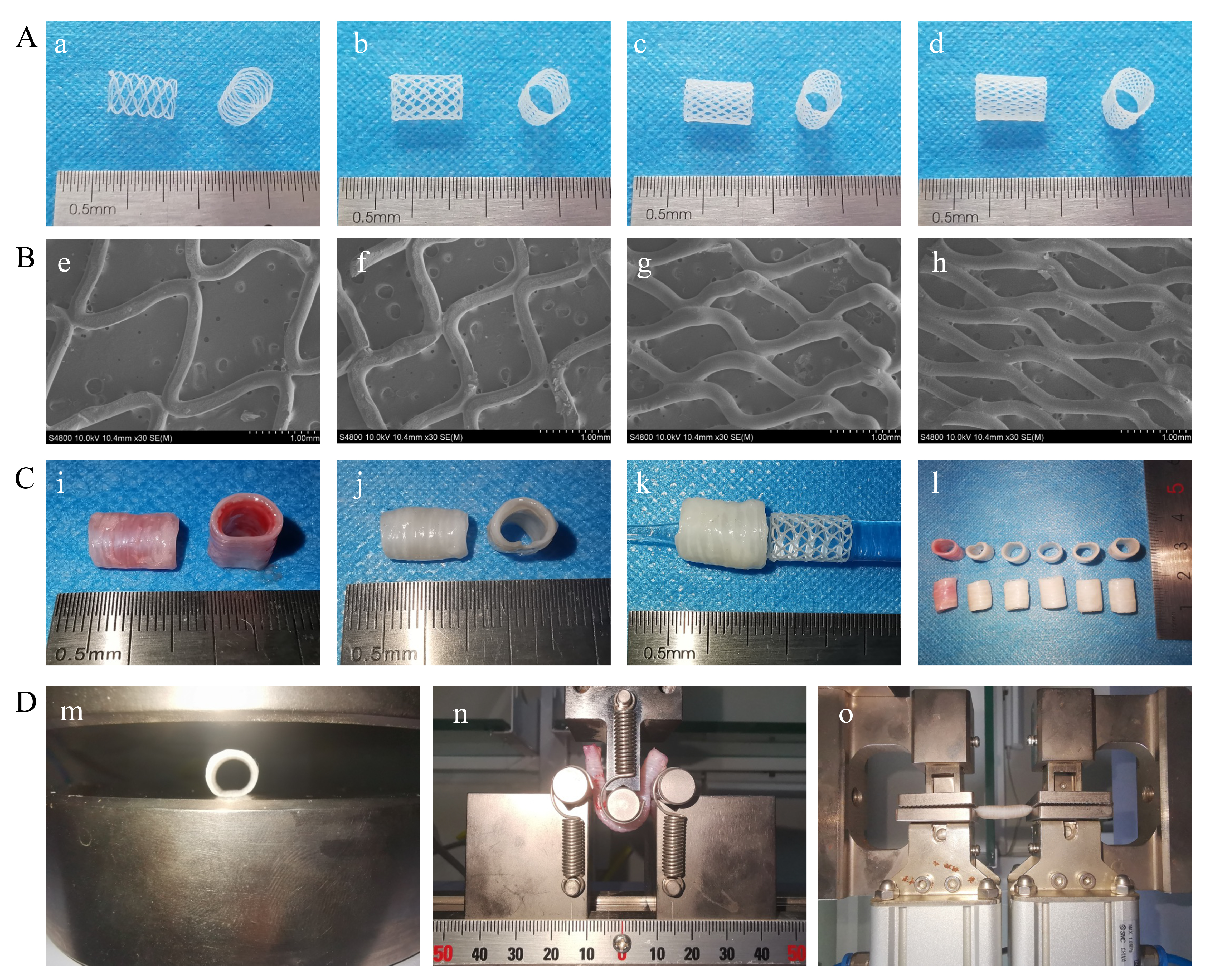

Supplement: Supplementary file 1 — Figure S1. Morphology of 3D printed stents and VADT/PCL hybrid grafts. (A) Macroscopic images of 3D printed (a) PCL‐10, (b) PCL‐20, (c) PCL‐30, and (d) PCL‐40 stents. (B) SEM images of the 3D printed (e) PCL‐10, (f) PCL‐20, (g) PCL‐30, and (h) PCL‐40 stents. (C) Macroscopic images of the (i) native trachea, (j) VADT, (k) assembly process of VADT/PCL hybrid grafts, and (l) different group of grafts (from left to right; native, VADT, VADT/PCL‐10, VADT/PCL‐20, VADT/PCL‐30, and VADT/PCL‐40 tracheal grafts). (D) Macroscopic images of (m) compressive, (n) three‐point bending, and (o) tensile tests. [file BTM2-8-e10534-s002.jpg]

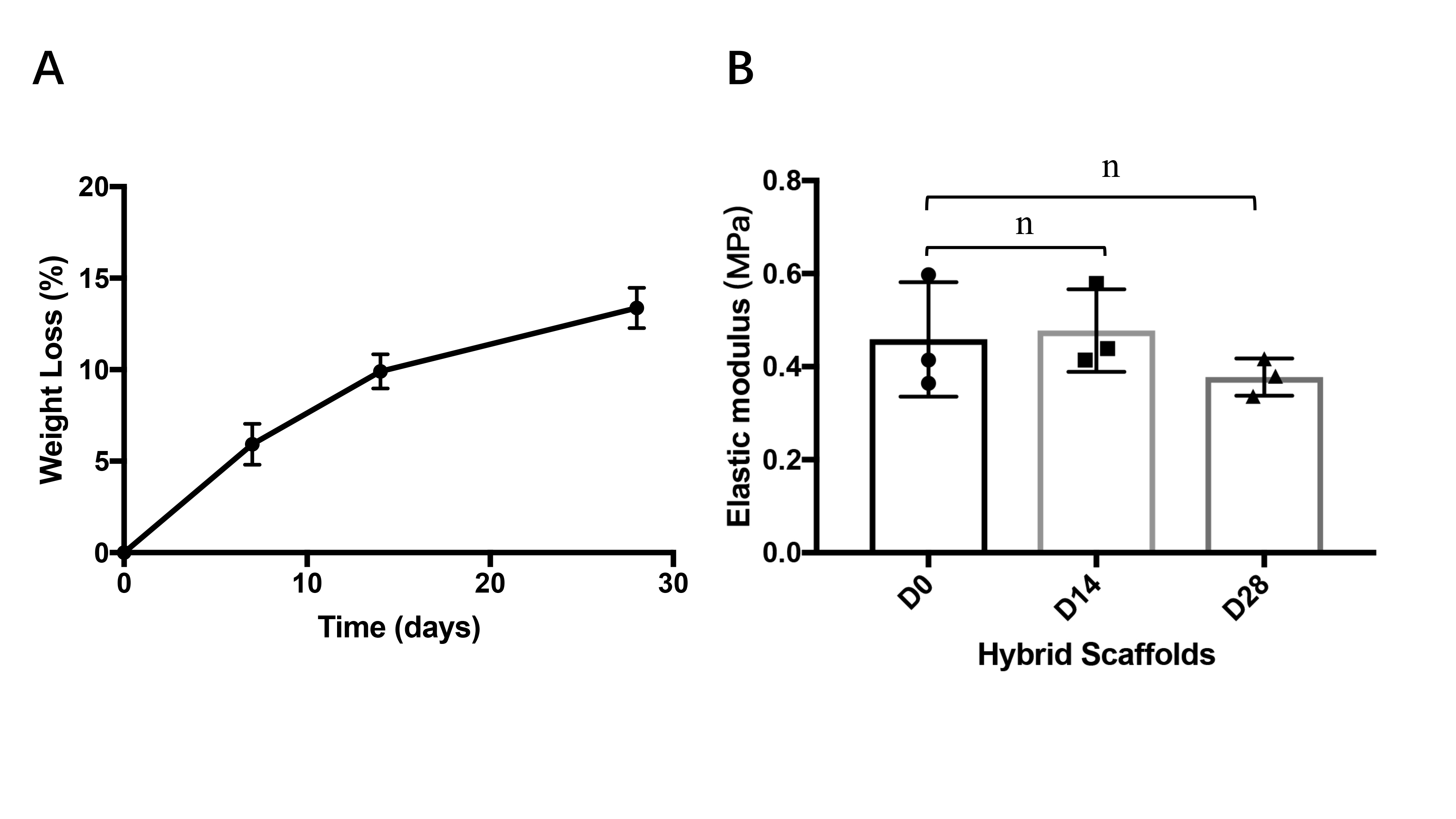

Supplement: Supplementary file 2 — Figure S2. Physical characterization of the VADT/PCL‐20 scaffolds. (A) Degradation characterization of the scaffolds (n = 3, p < 0.05). (B) Tensile mechanical properties of the scaffolds during degradation (n = 3, p > 0.05). [file BTM2-8-e10534-s001.jpg]

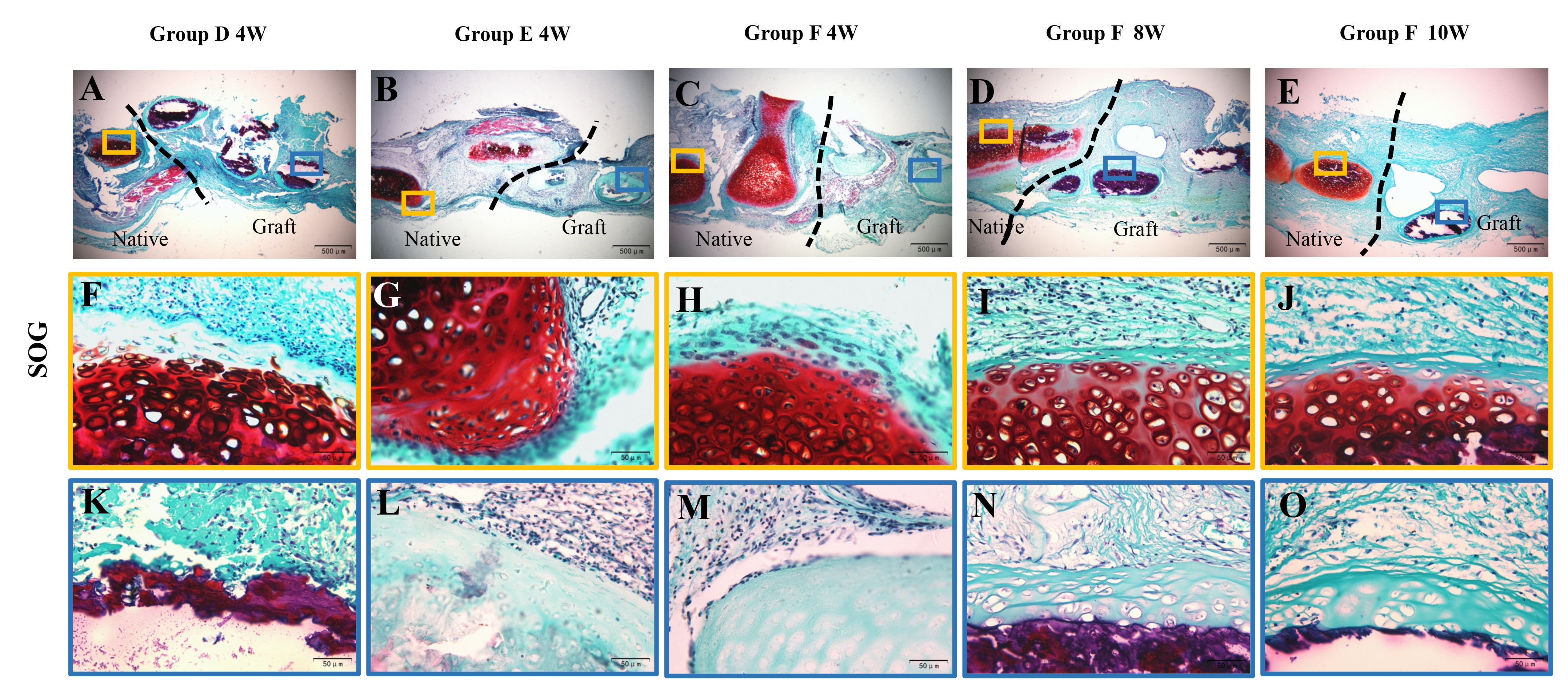

Supplement: Supplementary file 3 — Figure S3. SOG staining showing the histology of grafts for recipients surviving more than 4 weeks. (F–J) Cartilage area of native tracheae. Scale bars = 50 μm. (K–O) Cartilage area of grafts. (A–E) Scale bars = 500 μm. [file BTM2-8-e10534-s003.jpg]
