# Supplementary material for: Biomimetic in situ tracheal microvascularization for segmental tracheal reconstruction in one‐step
Source: Bioeng Transl Med. 2023 May 3;8(4):e10534. doi: 10.1002/btm2.10534 (PMC10354772; doi:10.1002/btm2.10534)
Supplement: Supplementary file 4 — Data S1. Supporting Information. [file BTM2-8-e10534-s004.docx]

**Supplemental Materials**


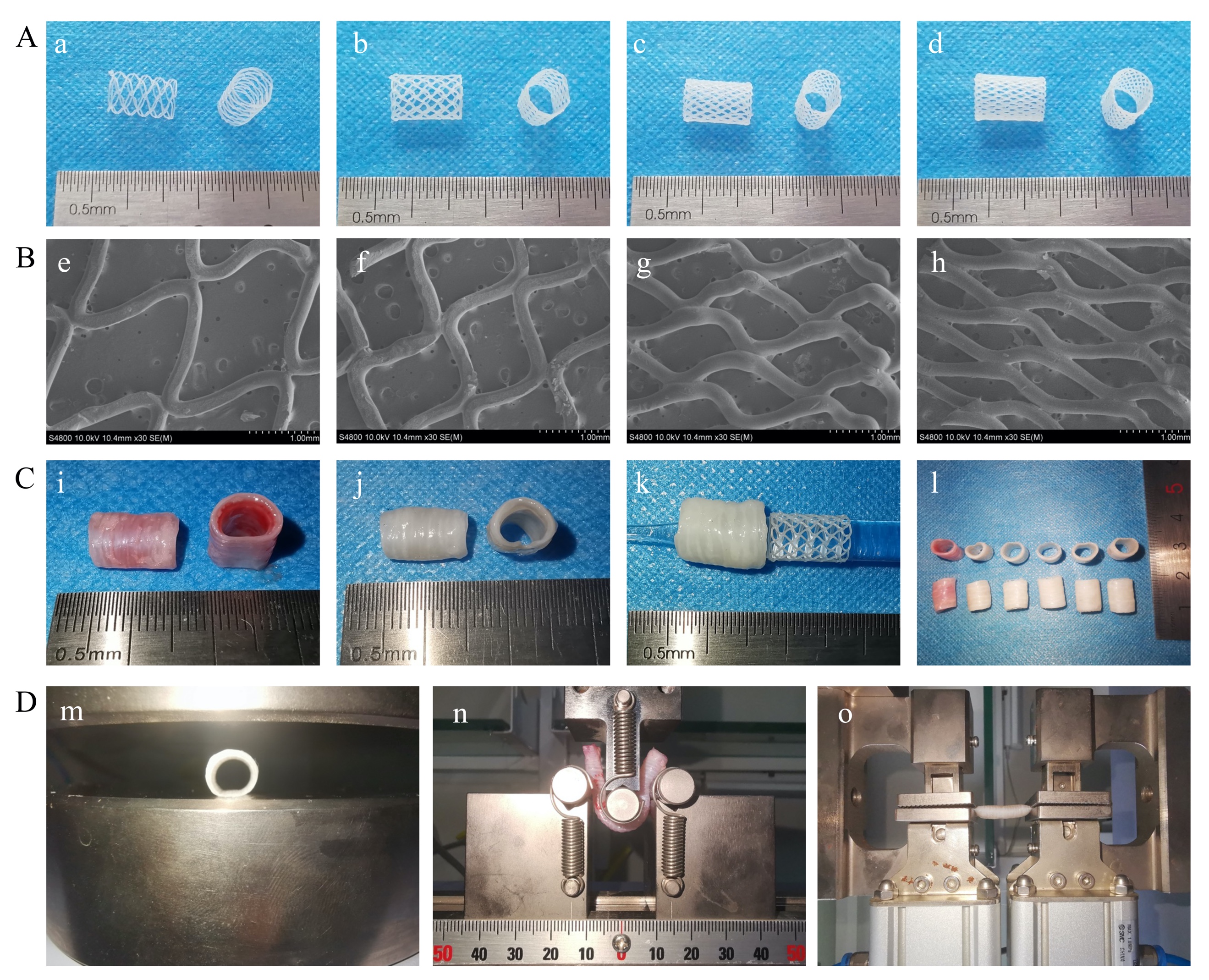


**Supplementary Figure 1. Morphology of 3D printed stents and VADT/PCL hybrid grafts.** (A) Macroscopic images of 3D printed (a) PCL-10, (b) PCL-20, (c) PCL-30, and (d) PCL-40 stents. (B) SEM images of the 3D printed (e) PCL-10, (f) PCL-20, (g) PCL-30, and (h) PCL-40 stents. (C) Macroscopic images of the (i) native trachea, (j) VADT, (k) assembly process of VADT/PCL hybrid grafts, and (l) different group of grafts (from left to right; native, VADT, VADT/PCL-10, VADT/PCL-20, VADT/PCL-30, and VADT/PCL-40 tracheal grafts). (D) Macroscopic images of (m) compressive, (n) three-point bendingand, and (o) tensile tests.

**
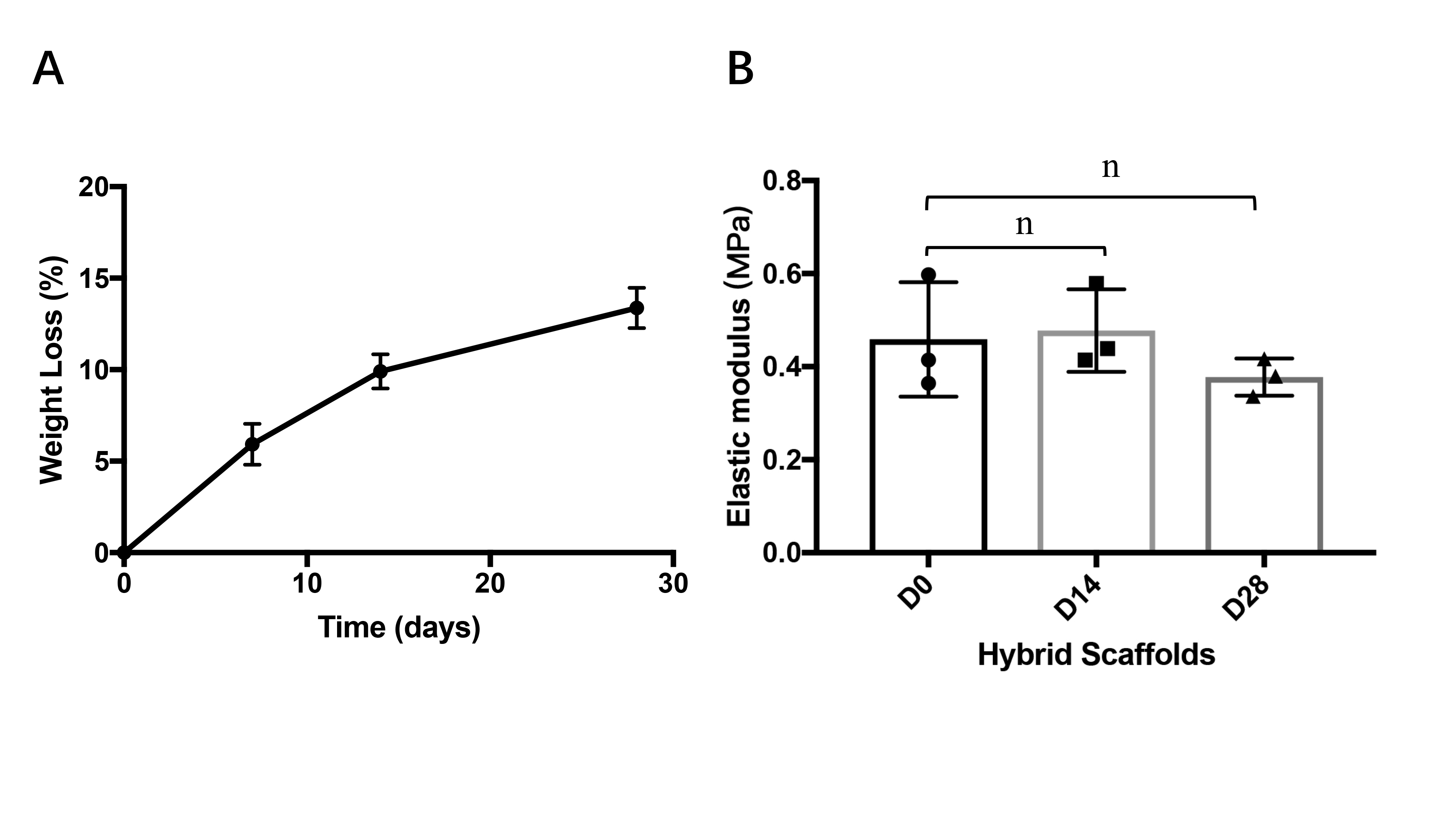
Supplementary Figure 2. Physical characterization of the VADT/PCL-20 scaffolds.** (A) Degradation characterization of the scaffolds (n = 3, *P* < 0.05). (B) Tensile mechanical properties of the scaffolds during degradation (n = 3, *P* > 0.05)


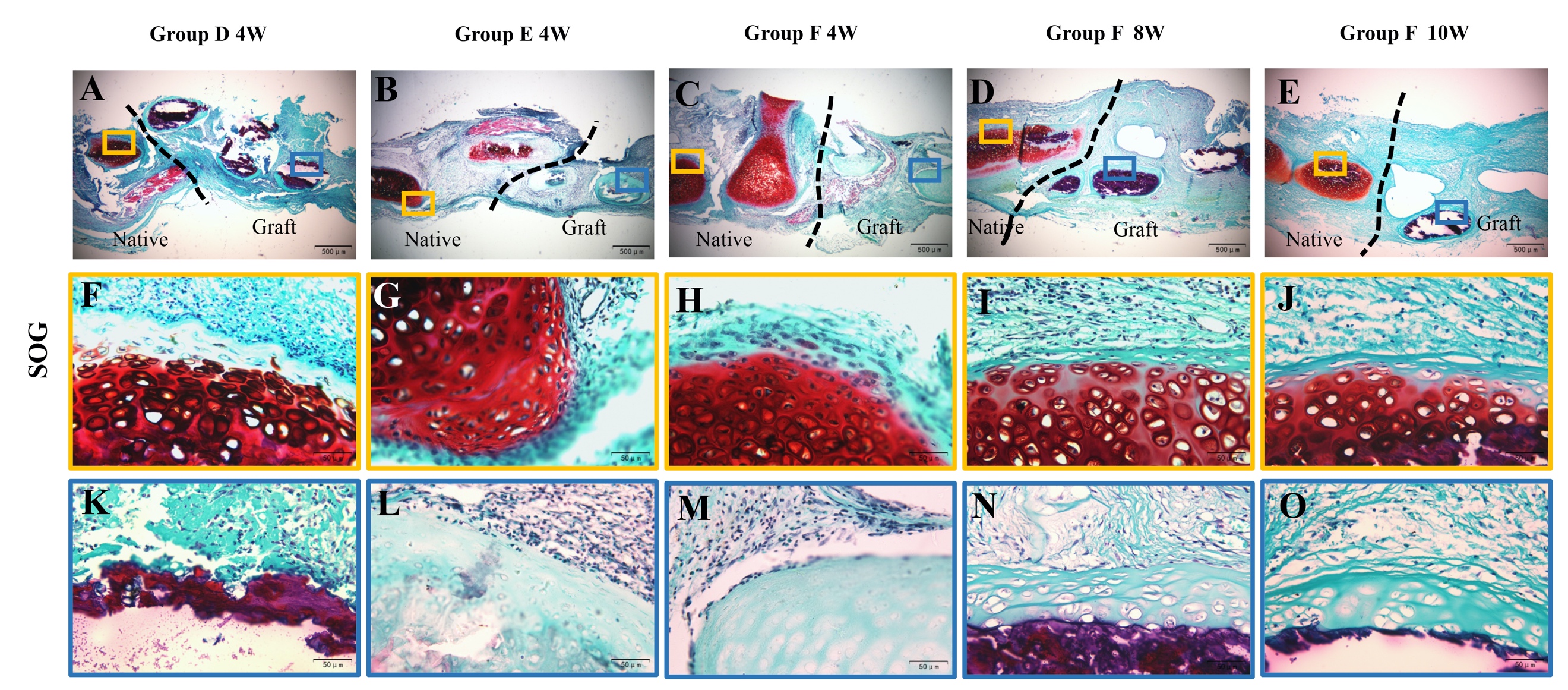


**Supplementary Figure 3. SOG staining showing the histology of grafts for recipients surviving more than 4 weeks.** (F–J) Cartilage area of native tracheae. Scale bars = 50 µm. (K–O) Cartilage area of grafts. (A–E) Scale bars = 500 µm.

**Supplementary Table 1. Biomechanical performance testing of each group.**

| Tests | | Groups | n1 | n2 | n3 | Average | Stdev |
| --- | --- | --- | --- | --- | --- | --- | --- |
| Compressive | Load of 50% ccompression of lumen | Native | 0.375 | 0.322 | 0.360 | 0.35 | 0.03 |
|  |  | VADT | 0.044 | 0.031 | 0.080 | 0.05 | 0.03 |
|  |  | VADT/PCL-10 | 0.261 | 0.177 | 0.232 | 0.22 | 0.04 |
|  |  | VADT/PCL-20 | 0.238 | 0.416 | 0.220 | 0.29 | 0.11 |
|  |  | VADT/PCL-30 | 0.196 | 0.295 | 0.261 | 0.25 | 0.05 |
|  |  | VADT/PCL-40 | 0.328 | 0.224 | 0.382 | 0.31 | 0.08 |
|  | Elastic modulus of between 20% and 80% max displacement | Native | 0.150 | 0.267 | 0.200 | 0.21 | 0.06 |
|  |  | VADT | 0.023 | 0.017 | 0.055 | 0.03 | 0.02 |
|  |  | VADT/PCL-10 | 0.129 | 0.140 | 0.120 | 0.13 | 0.01 |
|  |  | VADT/PCL-20 | 0.106 | 0.211 | 0.336 | 0.22 | 0.12 |
|  |  | VADT/PCL-30 | 0.126 | 0.182 | 0.159 | 0.16 | 0.03 |
|  |  | VADT/PCL-40 | 0.198 | 0.123 | 0.141 | 0.15 | 0.04 |
| Three-point bending | Load of displacement in 15mm | Native | 0.171 | 0.306 | 0.175 | 0.25 | 0.07 |
|  |  | VADT | 0.001 | 0.002 | 0.007 | 0.003 | 0.003 |
|  |  | VADT/PCL-10 | 0.095 | 0.110 | 0.178 | 0.13 | 0.04 |
|  |  | VADT/PCL-20 | 0.472 | 0.433 | 0.308 | 0.40 | 0.09 |
|  |  | VADT/PCL-30 | 0.932 | 0.460 | 0.838 | 0.74 | 0.25 |
|  |  | VADT/PCL-40 | 0.518 | 0.378 | 0.639 | 0.51 | 0.13 |
|  | Elastic modulus of between 20% and 80% max load | Native | 0.136 | 0.187 | 0.112 | 0.15 | 0.04 |
|  |  | VADT | 0.010 | 0.003 | 0.004 | 0.005 | 0.003 |
|  |  | VADT/PCL-10 | 0.051 | 0.053 | 0.111 | 0.07 | 0.03 |
|  |  | VADT/PCL-20 | 0.446 | 0.377 | 0.272 | 0.37 | 0.09 |
|  |  | VADT/PCL-30 | 1.174 | 1.350 | 0.913 | 1.15 | 0.22 |
|  |  | VADT/PCL-40 | 0.768 | 0.368 | 0.588 | 0.57 | 0.20 |
| Tensile | Load of breaking point | Native | 6.073 | 5.362 | 10.03 | 7.15 | 2.52 |
|  |  | VADT | 2.829 | 2.052 | 1.629 | 2.17 | 0.61 |
|  |  | VADT/PCL-10 | 7.613 | 7.758 | 8.179 | 7.85 | 0.29 |
|  |  | VADT/PCL-20 | 13.55 | 21.29 | 18.27 | 17.70 | 3.90 |
|  |  | VADT/PCL-30 | 27.83 | 28.97 | 32.84 | 29.88 | 2.62 |
|  |  | VADT/PCL-40 | 34.46 | 36.85 | 33.90 | 35.07 | 1.57 |
| Tensile | Elastic modulus of between 20% and 80% max load | Native | 3.962 | 1.584 | 1.604 | 2.38 | 1.37 |
|  |  | VADT | 0.642 | 0.312 | 0.313 | 0.42 | 0.19 |
|  |  | VADT/PCL-10 | 0.524 | 0.458 | 0.812 | 0.60 | 0.19 |
|  |  | VADT/PCL-20 | 2.127 | 2.217 | 2.021 | 2.12 | 0.10 |
|  |  | VADT/PCL-30 | 6.206 | 5.699 | 9.831 | 7.25 | 2.25 |
|  |  | VADT/PCL-40 | 10.55 | 4.561 | 5.556 | 6.89 | 3.21 |
| **Load (N), Elastic modulus(N/mm^2^).** | | | | | | | |

**Supplementary Table 2. The OD values of each group on day 1, 3, and 5.**

| Day | Group | n1 | n2 | n3 | n4 | Average | Stdev |
| --- | --- | --- | --- | --- | --- | --- | --- |
| 1 | VADT +EPCs | 0.328 | 0.340 | 0.312 | 0.330 | 0.33 | 0.01 |
|  | VADT | 0.067 | 0.044 | 0.067 | 0.070 | 0.06 | 0.01 |
|  | VADT/PCL-10 + EPCs | 0.326 | 0.310 | 0.277 | 0.314 | 0.31 | 0.02 |
|  | VADT/PCL-20 + EPCs | 0.329 | 0.311 | 0.259 | 0.288 | 0.30 | 0.03 |
|  | VADT/PCL-30 + EPCs | 0.219 | 0.268 | 0.296 | 0.315 | 0.28 | 0.04 |
|  | VADT/PCL-40 + EPCs | 0.219 | 0.288 | 0.316 | 0.299 | 0.28 | 0.04 |
| 3 | VADT +EPCs | 2.162 | 1.963 | 2.026 | 2.340 | 2.12 | 0.17 |
|  | VADT | 0.092 | 0.068 | 0.079 | 0.085 | 0.08 | 0.01 |
|  | VADT/PCL-10 + EPCs | 2.347 | 1.929 | 1.678 | 1.864 | 1.96 | 0.28 |
|  | VADT/PCL-20 + EPCs | 1.732 | 2.105 | 2.251 | 2.131 | 2.06 | 0.22 |
|  | VADT/PCL-30 + EPCs | 1.340 | 1.529 | 1.725 | 2.000 | 1.65 | 0.28 |
|  | VADT/PCL-40 + EPCs | 1.402 | 1.847 | 1.751 | 1.815 | 1.70 | 0.21 |
| 5 | VADT +EPCs | 2.477 | 2.05 | 2.888 | 3.313 | 2.68 | 0.54 |
|  | VADT | 0.111 | 0.102 | 0.086 | 0.087 | 0.10 | 0.01 |
|  | VADT/PCL-10 + EPCs | 2.082 | 1.68 | 2.595 | 2.536 | 2.22 | 0.43 |
|  | VADT/PCL-20 + EPCs | 1.765 | 2.805 | 1.764 | 2.388 | 2.18 | 0.51 |
|  | VADT/PCL-30 + EPCs | 2.284 | 1.656 | 1.835 | 1.652 | 1.86 | 0.30 |
|  | VADT/PCL-40 + EPCs | 1.921 | 2.273 | 1.921 | 2.215 | 2.08 | 0.19 |
